# Bitter tastants relax the mouse gallbladder smooth muscle independent of signaling through tuft cells and bitter taste receptors

**DOI:** 10.1038/s41598-024-69287-6

**Published:** 2024-08-08

**Authors:** Maryam Keshavarz, Anna-Lena Ruppert, Mirjam Meiners, Krupali Poharkar, Shuya Liu, Wafaa Mahmoud, Sarah Winterberg, Petra Hartmann, Petra Mermer, Alexander Perniss, Stefan Offermanns, Wolfgang Kummer, Burkhard Schütz

**Affiliations:** 1 Institute for Anatomy and Cell Biology, German Center for Lung Research, Justus Liebig University, Giessen, Germany; 2 Excellence Cluster Cardio-Pulmonary Institute, Justus Liebig University, Giessen, Germany; 3 Institute for Anatomy and Cell Biology, Philipps-University, Marburg, Germany; 4 Present Address: III. Department of Medicine, University Medical Center Hamburg-Eppendorf, Hamburg, Germany; 5 Department of Pharmacology, Max Planck Institute for Heart and Lung Research, Bad Nauheim, Germany; 6 Present Address: Anatomy and Cell Biology, Institute of Theoretical Medicine, Faculty of Medicine, University of Augsburg, Augsburg, Germany; 7 Present Address: Department of Anatomy, Faculty of Medicine, Jordan University of Science and Technology, Irbid, Jordan; 8 Present Address: Division of Allergy and Clinical Immunology, Department of Medicine, Jeff and Penny Vinik Center for Allergic Disease Research, Brigham and Women’s Hospital, Harvard Medical School, Boston, MA USA

**Keywords:** Cholecystokinin, Denatonium, Dextromethorphan, Taste transduction cascade, Transient receptor potential family member 5, Quinine, Cell biology, Molecular biology, Physiology

## Abstract

Disorders of gallbladder motility can lead to serious pathology. Bitter tastants acting upon bitter taste receptors (TAS2R family) have been proposed as a novel class of smooth muscle relaxants to combat excessive contraction in the airways and other organs. To explore whether this might also emerge as an option for gallbladder diseases, we here tested bitter tastants for relaxant properties and profiled *Tas2r* expression in the mouse gallbladder. In organ bath experiments, the bitter tastants denatonium, quinine, dextromethorphan, and noscapine, dose-dependently relaxed the pre-contracted gallbladder. Utilizing gene-deficient mouse strains, neither transient receptor potential family member 5 (TRPM5), nor the *Tas2r143/Tas2r135/Tas2r126* gene cluster, nor tuft cells proved to be required for this relaxation, indicating direct action upon smooth muscle cells (SMC). Accordingly, denatonium, quinine and dextromethorphan increased intracellular calcium concentration preferentially in isolated gallbladder SMC and, again, this effect was independent of TRPM5. RT-PCR revealed transcripts of *Tas2r108*, *Tas2r126*, *Tas2r135*, *Tas2r137*, and *Tas2r143*, and analysis of gallbladders from mice lacking tuft cells revealed preferential expression of *Tas2r108* and *Tas2r137* in tuft cells. A TAS2R143-mCherry reporter mouse labeled tuft cells in the gallbladder epithelium. An in silico analysis of a scRNA sequencing data set revealed *Tas2r* expression in only few cells of different identity, and from in situ hybridization histochemistry, which did not label distinct cells. Our findings demonstrate profound tuft cell- and TRPM5-independent relaxing effects of bitter tastants on gallbladder smooth muscle, but do not support the concept that these effects are mediated by bitter receptors.

## Introduction

The gallbladder stores and modifies bile between meals, and releases its content into the duodenum in response to feeding and the subsequent action of hormonal and neural signaling^1^. One critical factor in this dynamic process, contraction and relaxation of the gallbladder SMC layer, must be tightly controlled, and disturbances in gallbladder motility may lead to serious pathology, including inflammation and gallstone disease^2^. Endogenous factors known to contract the gallbladder smooth muscle include cholecystokinin (CCK) released from intestinal I-cells and nerve-derived acetylcholine (ACh), while e.g. bile salts and noradrenaline represent relaxing factors^2^. Recently, tuft cells, a minor cell type in the gallbladder epithelium, emerged as a further regulator of gallbladder tone. In the murine gallbladder, they responded to the bacterial metabolite, propionate, with a concomitant release of ACh and cysteinyl leukotrienes, which resulted in enhanced mucus secretion from gallbladder epithelial cells, and in smooth muscle contraction, respectively^3^.

In addition to such endogenous mediators of known cellular origin, bitter tasting substances have been identified as potent regulators of smooth muscle tone in other organs including airways and intestine^4^. Although some constrictor effects have been reported at select locations like the pulmonary vasculature^5^, relaxing effects prevail and exploiting bitter tastants as a new class of therapeutics to combat bronchoconstriction in asthma has been suggested^6^. Generally, these effects are attributed to activation of classical bitter taste receptors of the taste receptor type 2 (*Tas2r*) gene family, which, in mice, comprises about 35 functional genes^7^. Against initial expectation, their expression is not restricted to sensory (type II) cells of oropharyngeal taste buds, but they are widely expressed in extraoral tissues including smooth muscle, suggesting additional non-gustatory functions for these receptors^4,8–10^. Besides smooth muscle^11,12^, they have received particular attention in tuft cells of the respiratory tract and urethra as sensors of bacterial products, triggering innate immune effector mechanisms and protective neural reflexes^13–16^.

Downstream signaling has been originally characterized in type II taste cells of oropharyngeal taste buds^17,18^. Here, TAS2R are coupled to the α-subunit of gustducin (α-gust; gene name *Gnat3*). Upon binding of the ligand, the βγ-subunits are released, and a downstream signaling cascade is activated that comprises phospholipase C beta 2 (PLCβ2), inositol trisphosphate receptor 3 (IP_3_R3), and TRPM5^19^. During this process, the release of calcium ions from intracellular stores leads to an activation of the membrane-bound TRPM5, and this channel then allows an influx of sodium ions, ultimately resulting in membrane depolarization and release of transmitter substances^20^. Remarkably, this canonical taste transduction cascade is neither unique for taste receptor signaling, nor is it found in all cells expressing TAS2R family members. Tuft cells link this signaling pathway also to the succinate receptor SUCNR1 in the intestine and airways^21–24^ and to the free fatty acid receptor 2 in the gallbladder^3^, which here serve to detect microbial and danger-associated metabolites. In smooth muscle, on the other hand, expression of *Gnat3* and of *Trpm5* have been reported for the mouse gastric fundus^25^, but not for human airways, in which an alternative pathway comprising of the G protein G_αi1,2,3_, an unspecified phospholipase C beta, proteinase activated receptor 3, LIM kinase and cofilin shall mediate relaxation by TAS2R14^26,27^.

On this background, we hypothesized that prototypic bitter tastants exert relaxing effects, either directly on smooth muscle cells or secondary through action on tuft cells, and that TAS2R family members and components of the canonical downstream signaling cascade are expressed in the gallbladder. Relaxing properties of bitter tastants were tested on explanted gallbladders using appropriate genetic models to dissect the roles of tuft cells and of TRPM5, the central ion channel of the taste transduction cascade, in this process. In addition, we profiled the expression of *Tas2r* in the mouse gallbladder by RT-PCR, in silico analysis of available scRNA-seq data, in situ hybridization (ISH), and using a *Tas2r143* reporter mouse strain. The use of tuft cell-deficient mice allowed to determining the extent of *Tas2r* expression in non-tuft cells.

## Results

### Bitter tastants dose-dependently relax the pre-contracted gallbladder independent of the canonical taste transduction cascade

**Figure 1:**
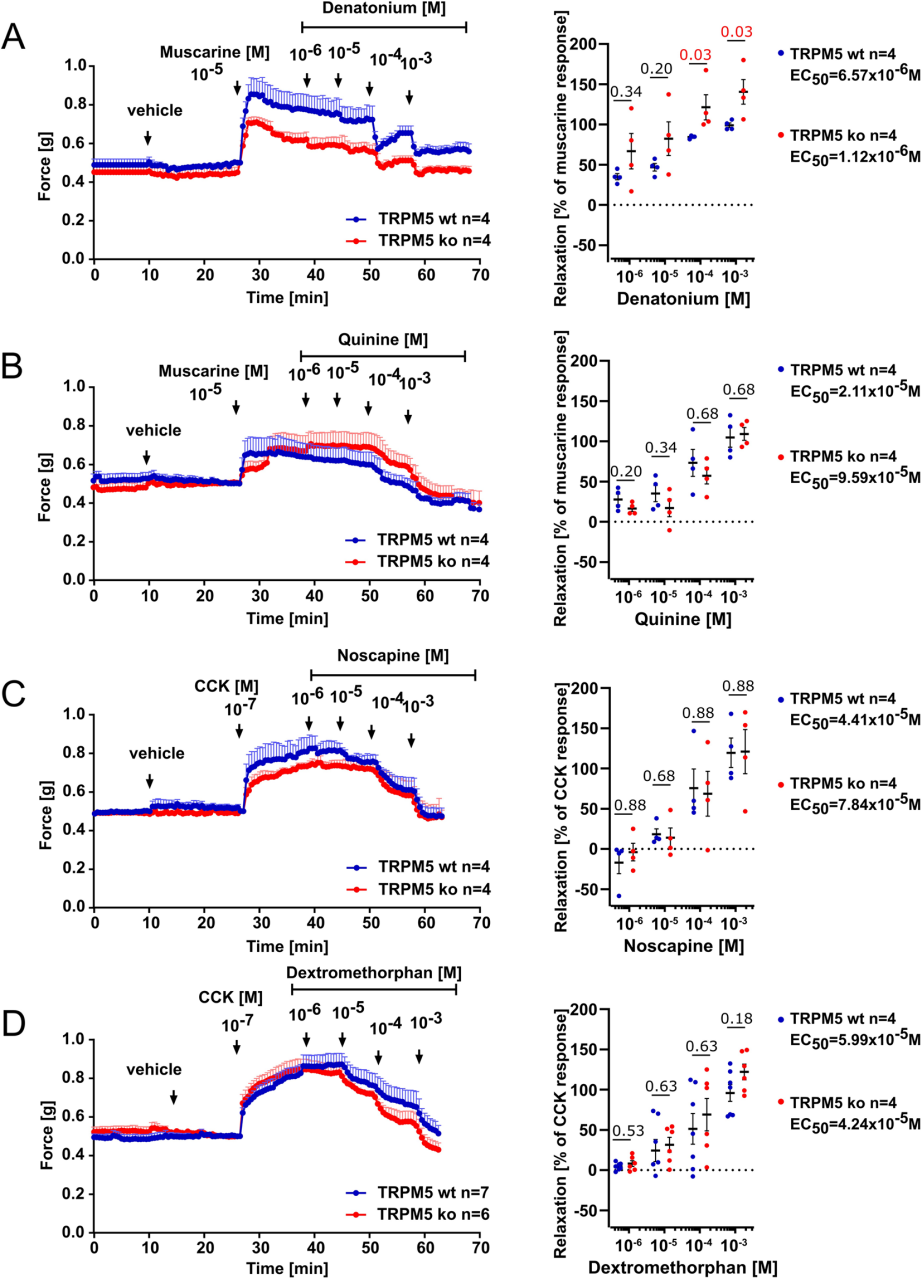
Relaxing effects of bitter taste receptor agonists on gallbladder smooth muscle tone. Organ bath force recordings traced the effects of increasing concentrations (1–1000 µM) of (**A**) denatonium, (**B**) quinine, (**C**) noscapine, and (**D**) dextromethorphan on gallbladder smooth muscle tone in *Trpm5*^+*/*+^ and *Trpm5*^*–/–*^ mice. Each data point represents the mean (n) ± SEM. Gallbladders were pre-contracted with muscarine (10 µM, A + B), or cholecystokinin (CCK) (0.1 µM, **C + D**), respectively. Note that EC_50_ were similar in both genotypes (*p* = 0.1544 for denatonium, *p* = 0.262 for quinine, *p* = 0.7656 for noscapine, *p* = 0.3554 for dextromethorphan). Scatter plots in (**A–D**) illustrate the maximum relaxation responses (%) induced by distinct bitter taste receptor agonists following the maximal contraction induced by muscarine or CCK. Whiskers represent means ± SEM across the dataset. Statistical analysis conducted via the Mann–Whitney-U-test and the exact p-values are reported.

We tested the capacity of typical bitter taste receptor agonists to relax gallbladder smooth muscle in an organ bath setup. Gallbladders were pre-contracted with either muscarine (10 µM) or CCK (100 nM). Both led to a rapid increase (0.30–0.35 g) in the contraction force, which then plateaued for at least 40 min (Sup. Fig. 1A) without being affected by further vehicle (ethanol or methanol) treatment (Sup. Fig. 2). To test for potential effects of bitter tastants, we employed compounds that previously have been described as relaxants in other organs^28,29^. Denatonium (1–1000 µM, Fig. 1A), agonist at e.g. TAS2R105, TAS2R108, TAS2R123, TAS2R135, TAS2R140 and TAS2R144^7,30^; quinine (1–1000 µM, Fig. 1B), an agonist at e.g. TAS2R108, TAS2R126, and TAS2R137^7^; noscapine (1–1000 µM, Fig. 1C), a broad TAS2R agonist acting upon human TAS2R14^31^ with unknown specificity for mouse TAS2Rs; and dextromethorphan (1–1000 µM, Fig. 1D), also a broadly active agonist acting upon human TAS2R1 and Tas2R10^5,28^, all relaxed the pre-contracted gallbladder in a concentration-dependent manner. All these relaxing effects occurred independent of signaling through TRPM5, because they were still present in *Trpm5*^*–/–*^ mice (Fig. 1). Notably, denatonium exhibited an increased relaxing effect at higher concentrations (100 µM and 1 mM) after maximal pre-contraction with muscarine in *Trpm5*^*–/–*^ mice (Fig. 1A). These experiments indicated that the tuft cell canonical taste transduction cascade was not involved in bitter tastant-induced gallbladder smooth muscle relaxation.

### The quinine-induced gallbladder smooth muscle relaxation occurs independent of TAS2R126, TAS2R135, and TAS2R143

**Figure 2:**
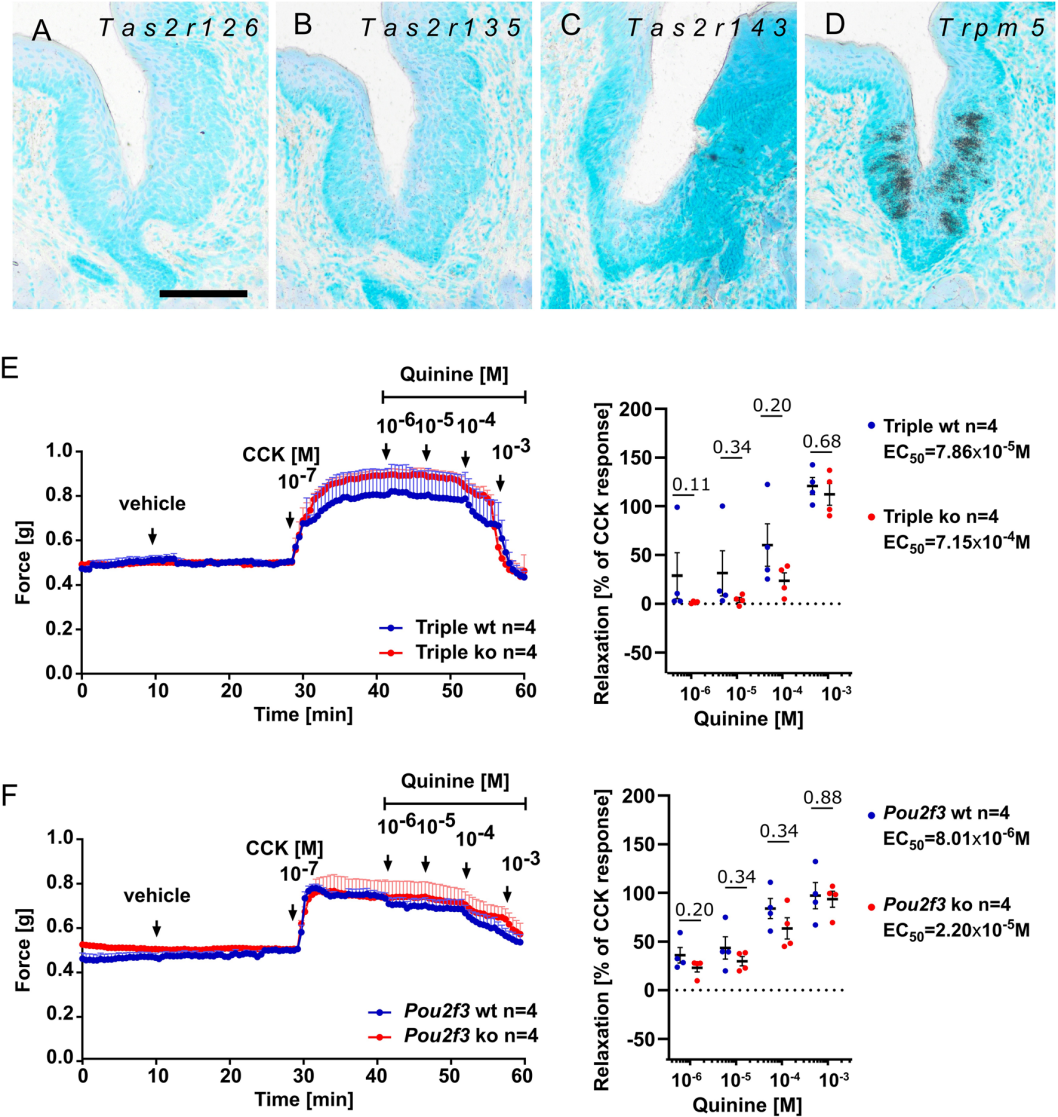
Quinine-induced gallbladder smooth muscle relaxation occurs independent of the *Tas2r143/135/126* gene cluster, and of the presence of tuft cells. (**A**–**D)** ISH experiments with gene-specific anti-sense riboprobes showing absence of *Tas2r126*, *Tas2r135*, and *Tas2r143* mRNA from vallate papilla taste buds in *Tas2r143/135/126* triple-knockout mice, while *Trpm5* mRNA is still present. Note that sense probes did not result in specific labeling (see Sup. Figs. 7 and 8). The scale bar in a equals 20 µm and accounts for (**A–D**). Organ bath recordings tracing the effects of increasing concentrations of quinine (1–1000 µM) on gallbladder smooth muscle tone in *Tas2r143/Tas2r135/Tas2r126*^+*/*+^ and *Tas2r143/Tas2r135/Tas2r126*^*–/–*^ mice (**E**), and in *Pou2f3*^+*/*+^ and *Pou2f3*^*–/–*^ mice (**F**). Each data point represents the mean (n) ± SEM. Gallbladders were pre-contracted with a single dose of CCK (0.1 µM). Scatter plots in (**E,F**) illustrate the maximum relaxation responses (%) triggered by quinine following the maximal contraction induced by CCK. Whiskers depict means ± SEM across the dataset. Notably, comparable relaxing effects were observed in both genotypes. The statistical analysis conducted using the Mann–Whitney-U-test and the exact p-values are reported.

Next, we analyzed if the quinine-induced relaxation was affected in mice that lack expression from the *Tas2r143/135/126* gene cluster encoding for one of the known quinine receptors (TAS2R126)^7^. Mice with genetic deletion of other quinine-responsive bitter receptors (TAS2R105, TAS2R108, TAS2R115, TAS2R137, TAS2R140, TAS2R144)^7^ are not available yet. In *Tas2r143/135/126* triple knockout mice^32^ expression of *Tas2r126*, *Tas2r135*, and *Tas2r143* was abolished, shown here for taste buds in the vallate papilla by ISH (Fig. 2A–D). In organ bath experiments, however, quinine (1–1000 µM) dose-dependently relaxed the pre-contracted gallbladder smooth muscle of triple knockout mice to a similar extent as in their corresponding wildtypes (Fig. 2E), indicating that TAS2R expression from this gene-cluster was not involved in this bitter tastant-induced relaxation. Also, the EC_50_ values were not significantly different between the genotypes (*p* = 0.1681, Fig. 2E).

### The quinine-induced gallbladder smooth muscle relaxation occurs independent of the presence of tuft cells

We then asked whether signaling through tuft cells was needed in the bitter tastant-induced relaxation, and tested the effect of quinine in *Pou2f3* knockout mice that lack gallbladder tuft cells^3,33^. Quinine (1–1000 µM) dose-dependently relaxed the pre-contracted gallbladder to a similar extent when compared to wildtype (Fig. 2F), indicating that gallbladder tuft cells were not involved in this bitter tastant-induced relaxation. The EC_50_ values were not significantly different between the genotypes (*p* = 0.2068, Fig. 2F).

### Denatonium, quinine, and dextromethorphan increase [Ca^2+^]_i_ in isolated gallbladder cells

**Figure 3:**
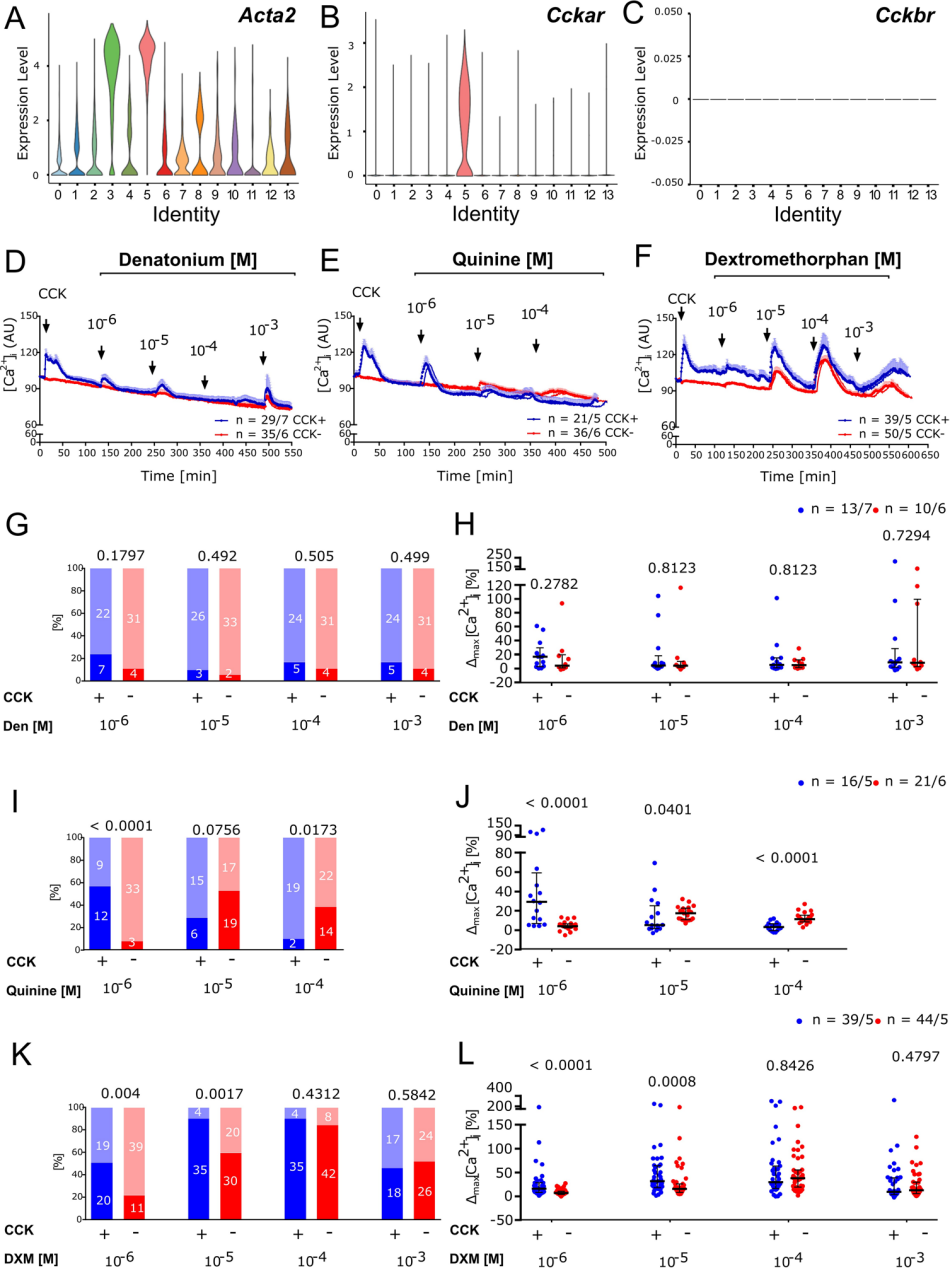
Bitter tastants increase [Ca^2+^]_i_ in isolated gallbladder cells. In silico analysis of a mouse gallbladder scRNA data set identified two cell clusters (#3 and 5) with high levels of *Acta2* expression (**A**). Cell cluster #5 represented smooth muscle cells, since it expressed the CCK receptor, *Cckar* (**B**), but not *Cckbr* (**C**). Based on CCK (1 µM) responsiveness, cells were grouped into CCK^+^ (presumably SMC) and CCK^–^ (all other) cells. Confocal laser scanning recording of fluorescence intensity of the calcium indicator Fluo-4 was expressed in arbitrary units (AU) over time in response to increasing concentrations of (**D**) denatonium (Den), (**E**) quinine, and (**F**) dextromethorphan (DXM), respectively, after CCK (1 µM) as an initial test stimulus. All drugs were added under continuous flow in the chamber, so that indicated concentrations were reached initially and then washed out. Mean ± SEM, *n* refers to number of cells/number of gallbladders from which they were taken. (**G,I,K**) Relative frequencies of responders (> 10% increase in [Ca^2+^]_i_; dark column) and non-responders (light column) to increasing concentrations of Den, quinine, and DXM in the experiments depicted in (**A–C**). Frequencies of responders among CCK^+^ cells were compared to CCK^–^ cells by chi-square test, *p*-values indicated above the columns; *n* as in (**A–C**), absolute numbers indicated in the columns. (**H,J,L**) Maximum increase in [Ca^2+^]_i_ in responders (cells that exhibited > 10% increase in [Ca^2+^]_i_ to at least one concentration of the respective bitter tastant); extent of reaction in CCK^+^ cells is compared to that in CCK^–^ cells by Mann–Whitney-U-test, *p*-values are indicated; *n* refers to number of cells/number of gallbladders from which they were taken.

In human airways, denatonium and quinine have been proposed to be potent bronchodilators, and this effect was paradoxically associated with increases in [Ca^2+^]_i_ in airway smooth muscle cells in vitro^12^. In mice, on the other hand, both Ca^2+^-rising and Ca^2+^-lowering effects of bitter tastants have been described, with the airway smooth muscle cells being at rest or contracted, respectively^34^. Along this line we here analyzed the effect of the three bitter tastants, denatonium, quinine, and dextromethorphan on [Ca^2+^]_i_ in dissociated gallbladder cells loaded with the Ca^2+^-indicator, Fluo-4. Vehicle alone (methanol 0.5 × 10^–3^% for denatonium; ethanol 0.5 × 10^–3^% M for dextromethorphan and quinine) evoked fluctuations in [Ca^2+^]_i_ of less than 10% (data not shown). Therefore, we took a response of 10% change at minimum to define cells as responders to a test stimulus. Cells responsive to 1 µM CCK (CCK^+^) were considered as smooth muscle cells (*Acta2*-expressing), since expression of the CCK receptor A (*Cckar*) was confined to cluster #5 in an in silico-analysis of a scRNA-seq data set (GSM5888949 https://krupalipoharkar.shinyapps.io/gallbladder_data/^33^), while the *Cckbr* subtype was not expressed. An additional *Acta2*^*high*^ cell population (cluster #3) did not express CCK receptors and probably represented vascular smooth muscle cells (Fig. 3A–C).

Both CCK^+^ and CCK^–^ cells reacted with a brief increase in [Ca^2+^]_i_ to denatonium and dextromethorphan over a concentration range from 10^–6^ to 10^–3^ M, and to quinine over a concentration range from 10^–6^ to 10^–4^ M (Fig. 3D–F). Reactivity declined at higher concentrations when agonists were given successively, indicating desensitization. This was further supported for denatonium and quinine by experiments in which agonists were given at highest dose directly after the initial CCK stimulus, showing stronger responses than in the cumulative administration series (Sup. Fig. 3A, B). In case of dextromethorphan at 10^–3^ M, however, such a desensitizing effect was not observed (Sup. Fig. 3C). The proportion of responders (≥ 10% increase) to denatonium varied from 5 to 25% over the tested concentration range without significant differences between CCK^+^ and CCK^–^ cells (Fig. 3G), neither were there significant differences in the magnitude of the increase in [Ca^2+^]_i_ between various denatonium concentration, nor between responding CCK^+^ and CCK^–^ cells (Fig. 3H). These data indicated non-specific effects of denatonium and thus this bitter taste stimulus was not further applied. In contrast, responders to the lowest concentration (10^–6^ M) of quinine and DXM were much more frequent (about 50%) than those to denatonium in the CCK^+^ population, and significantly less responders were found in the CCK^–^ group (Fig. 3I, K). Also, the magnitude of [Ca^2+^]_i_ increase was significantly higher in CCK^+^ than in CCK^–^ cells at this bitter tastant concentration (Fig. 3J, L). In the cumulative administration experiments, responsiveness of CCK^+^ and CCK^–^ cells swapped with increasing concentration of quinine, with significantly more responders and higher reactivity of CCK^–^ cells at 10^–5^ and 10^–4^ M (Fig. 3J).

**Figure 4:**
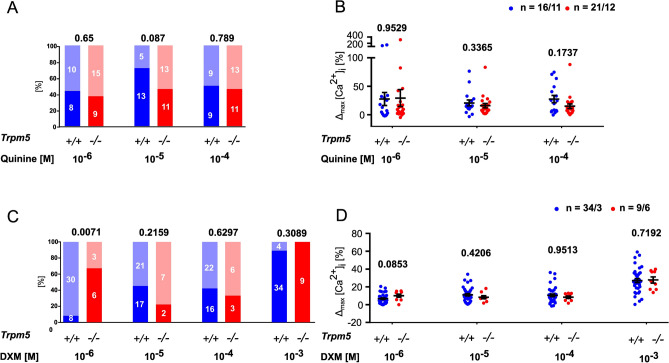
TRPM5 is not involved in [Ca^2+^]_i_ responses of isolated gallbladder smooth muscle cells (CCK^+^) to quinine and dextromethorphan. Confocal laser scanning recordings of fluorescence intensity of the calcium indicator Fluo-4 fluorescence in CCK^+^ cells. (**A,C**) Relative frequencies of responders (> 10% increase in [Ca^2+^]_i_; dark column) and non-responders (light column) to increasing concentrations of quinine and dextromethorphan (DXM) in cells from *Trpm5* wildtype (*Trpm5*^+*/*+^) and gene-deficient (*Trpm5*^*–/–*^) mice. Chi-square test shows no significant difference between genotypes, *p*-values are indicated; absolute numbers of cells indicated in the columns. (**B,D**) Maximum increase in [Ca^2+^]_i_ in responders (cells that exhibited > 10% increase in [Ca^2+^]_i_ to at least one concentration of the respective bitter tastant) to (**B**) quinine and (**D**) dextromethorphan (DXM); extent of reaction is not significantly different between genotypes (*Trpm5*^+*/*+^ versus *Trpm5*^*–/–*^); Mann–Whitney-U-test, *p*-values are indicated; *n* refers to number of cells/number of gallbladders from which they were taken.

To analyze whether quinine and dextromethorphan exerted their effects through the canonical taste transduction signaling cascade, experiments were conducted with the *Trpm5* gene-deficient mouse line. Notably, WT mice from this line (*Trpm5*^+*/*+^) were overall less reactive in calcium imaging experiments than C57BL/6J mice, including their response to CCK at 10^–6^ M (Sup. Fig. 4). Genetic deletion of *Trpm5* did not further affect the CCK response (Sup. Fig. 4), and neither the rate of responders nor the magnitude of the increase in [Ca^2+^]_i_ evoked by dextromethorphan and quinine was reduced in *Trpm5*^*–/–*^ compared to *Trpm5*^+*/*+^ mice both in CCK^+^ (Fig. 4) and CCK^–^ (Sup. Fig. 5) cells. Collectively, these data show that quinine and dextromethorphan increase [Ca^2+^]_i_ preferentially, but not exclusively, in gallbladder smooth muscle cells (CCK^+^), but this effect was independent of the canonical taste transduction signaling cascade involving TRPM5.

### Bitter taste receptors are differentially expressed in gallbladder and vallate papilla

**Figure 5:**
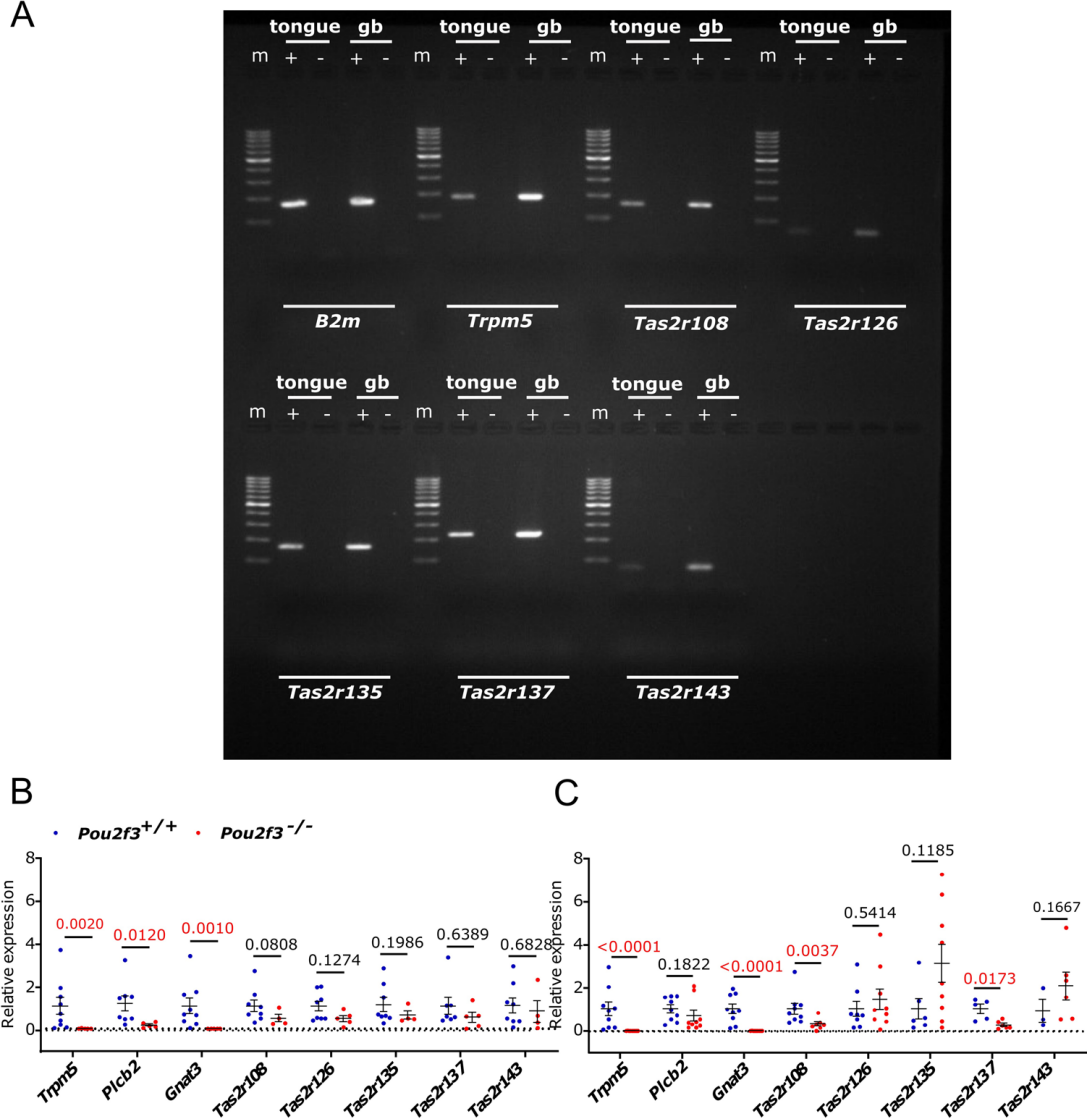
RT-PCR analysis of *Tas2r* gene expression in tongue and gallbladder showing those gene family members, which were consistently found in gallbladder samples (cf. Sup. Table 2). (**A**) Representative agarose gel pictures from RT-PCR experiments in the presence (+) or absence (−) of reverse transcriptase are displayed. *B2m* and *Trpm5* analysis served as positive controls for the integrity of the samples. m, 100 bp DNA ladder; *gb* gallbladder. (**B**) Vallate papilla, and (**C**) gallbladder RT-PCR on samples taken from *Pou2f3*^+*/*+^ mice, and from mice that lack type II taste cells and tuft cells (*Pou2f3*^*–/–*^). *n* = 4–9, The data was analyzed using Mann–Whitney-U-test, and the exact *p*-values are reported.

Our findings of bitter tastant-induced smooth muscle relaxation prompted us to identify their molecular targets and the corresponding cell type identity. The mouse gallbladder mucosal epithelium harbors chemosensory tuft cells^3,35–37^. Since both, tuft cell-dependent paracrine and cell-autonomous autocrine actions of TAS2R agonists have been described, e.g. in lung and gut^9^, we here assessed the presence and cellular localization of *Tas2r* gene expression in the mouse gallbladder. First, the expression pattern of all 35 mouse *Tas2r* genes was studied in whole gallbladder preparations taken from C57BL/6mice by RT-PCR, and compared with samples from vallate papilla. While all *Tas2r* family members were found to be expressed in the vallate papilla, a limited expression profile was noted in the gallbladder. Here, especially *Tas2r108*, *Tas2r126*, *Tas2r135*, *Tas2r137* and *Tas2r143* were consistently detected, whereas transcripts of *Tas2r138* were found only in half of the gallbladder samples (3/6) and all others even less frequent or not at all (Sup. Table 2 and Fig. 5A). To estimate the contribution of tuft and type II taste cells to *Tas2r* expression in the gallbladder and tongue, respectively, we compared samples from *Pou2f3*^*–/–*^ mice, which lack tuft and type II taste cells^38^, with those from their wildtype littermates (*Pou2f3*^+*/*+^) by real-time RT-PCR. In both the vallate papilla (Fig. 5B) and in the gallbladder (Fig. 5C), absence of tuft/taste cells resulted in complete loss of detectable expression of *Trpm5* and *Gnat3*, coding for two components of the canonical taste transduction signal cascade. The expression of *Plcb2*, on the other hand, was lost in the vallate papilla, but not in the gallbladder, indicating expression in additional cell types beyond the tuft cell lineage in this organ. Similarly, expression levels of *Tas2r126*, *Tas2r135*, and *Tas2r143* were found unchanged between the two genotypes, both in tongue and in gallbladder, while *Tas2r108* and *Tas2r137* expression levels were largely reduced in gallbladder samples from *Pou2f3*^*–/–*^ mice (Fig. 5B, C).

**Figure 6:**
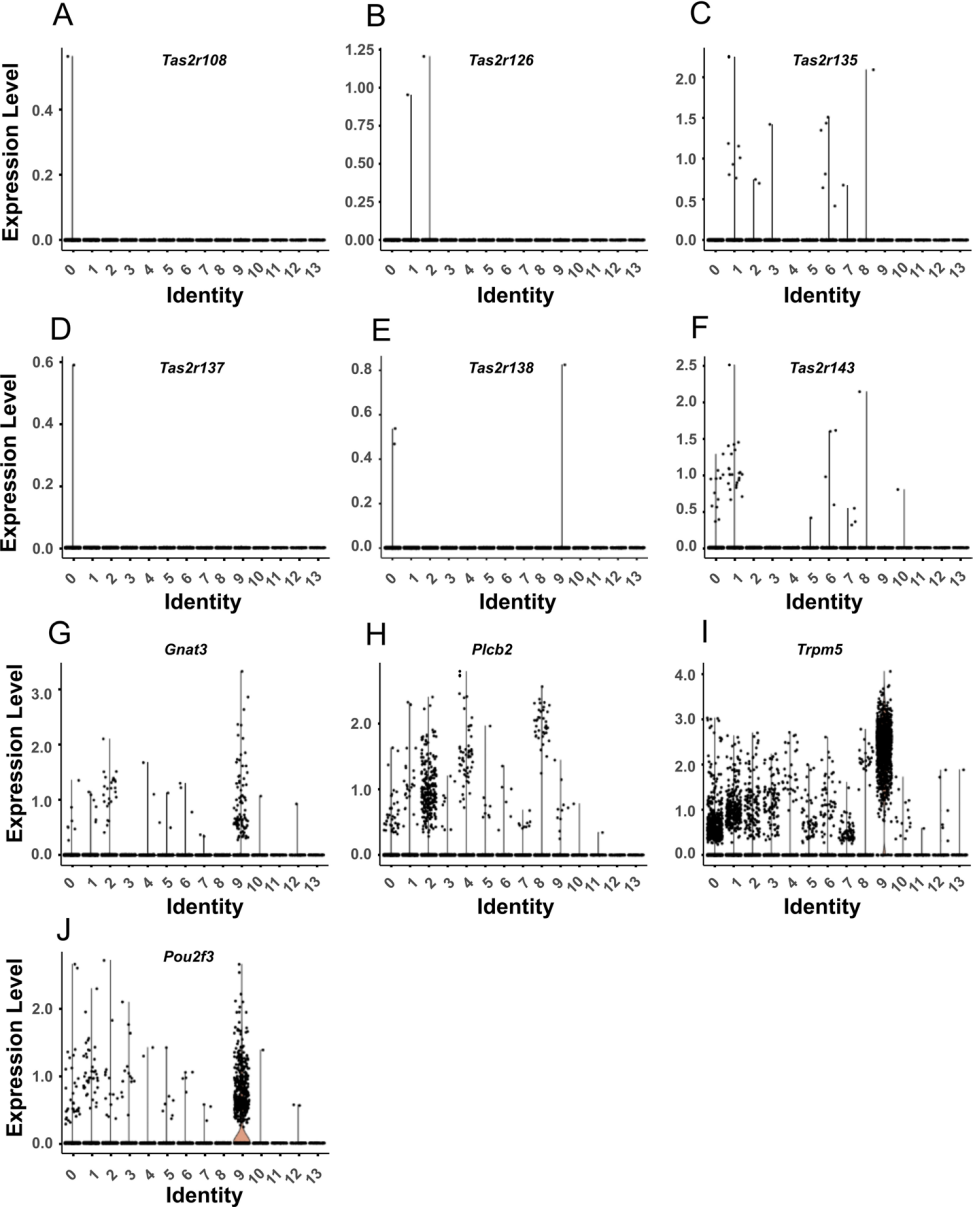
*Tas2r* and canonical taste transduction pathway expression patterns in mouse gallbladder. In silico analysis of single-cell RNA sequencing data of murine gallbladder cells (data set GSM5888949^33^). Unbiased clustering revealed 14 distinct cell clusters (0–13), among which cluster #9 represented tuft cells. Violin plots show expression of *Tas2r* in distinct cell clusters **(A-F)**, *Gnat3* **(G)**, *Plcb2* **(H)**, *Trpm5* **(I)** and *Pou2f3* **(J)**. Expression of *Tas2r108* was restricted to cluster #0 (epithelium; 1 out of 9695 cells). *Tas2r126* to clusters #1 (fibroblast; 1/7771) and 2 (mesenchyme; 1/3263). *Tas2r135* to cluster #1 (7/7771), #2 (2/3263), #3 (SMC; 1/2457), #6 (endothelium; 4/1266), #7 (undefined; 1/1179), and #8 (mesenchyme; 1/1065). *Tas2r137* to cluster #0 (1/9695). *Tas2r138* to #0 (2/9695) and #9 (tuft cell; 1/852). Tas2r143 to #0 (12/9695), #1 (17/7771), #5 (SMC; 1/1359), #6 (4/1266), #7 (3/1179), #8 (1/1065), and #10 (lymphatic endothelium; 1/275).

We further analyzed a publicly available single cells RNA-sequencing data set (GSM5888949 https://krupalipoharkar.shinyapps.io/gallbladder_data/^33^) of mouse gallbladder for the expression of the canonical taste transduction pathway and of *Tas2r* family members. Unbiased clustering revealed 14 distinct cell clusters, among which cluster #9 represented tuft cells, identified by *Pou2f3* expression (Fig. 6), and clusters #3 and #5 smooth muscle cells, identified by high *Acta2* expression (Fig. 3A–C). In this data set, *Tas2r* expression was restricted to only those 6 family members, which also had been found consistently in the previous RT-PCR screen (*Tas2r108*, *Tas2r126*, *Tas2r135*, *Tas2r137*, *Tas2r143*), or in 50% of samples (*Tas2r138*). In all these cases only very few cells or even only single hits within a cluster were noted. Specifically, in the smooth muscle cell populations, only one *Tas2r143* expressing cell was found in the *Cckar*^+^ (CCK-responsive) population (cluster #5) and only one *Tas2r135* expressing cell in the *Cckar*^*-*^ population (cluster #3) (Fig. 6).

Among the crucial elements of the canonical taste transduction pathway, expression of the taste-specific G protein *Gnat3* and of the cation channel *Trpm5* was enriched in tuft cells, although not being fully exclusive for this cluster (Fig. 6H, I), although real-time RT-PCR had not revealed detectable levels in *Pou2f3*^*–/–*^ mice (Fig. 5C). Specifically, some *Trpm5* hits were found in both smooth muscle cell populations (Fig. 6I). To determine, whether this low expression might result in detectable TRPM5 protein, gallbladders from a *Chat*^*BAC*^-GFP tuft cell-reporter mouse strain were subjected to TRPM5-immunolabeling. In support of the RT-PCR data, TRPM5-immunoreactivity was restricted to the cholinergic, i.e. *Chat*^*BAC*^-eGFP-immunoreactive tuft cells, but not seen in α-smooth-muscle-actin-immunoreactive smooth muscle cells (Sup. Fig. 6). In accordance with the real-time RT-PCR data on *Pou2f3*^*–/–*^ mice, expression of *Plcb2* showed no preference for tuft cells in the sequencing data set but was scattered across gallbladder cell populations with particularly few positive smooth muscle cells (Fig. 6H).

**Figure 7:**
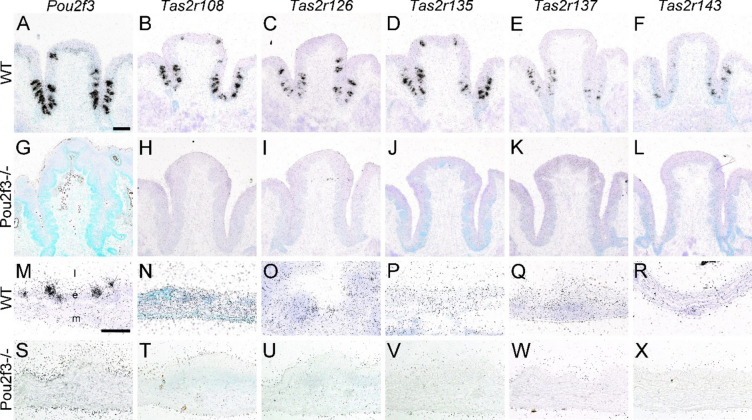
*Tas2r* expression patterns in mouse taste buds and gallbladder. In the vallate papilla from *Pou2f3*^+*/*+^ mice, expression of *Pou2f3* (**A**), *Tas2r108* (**B**), *Tas2r126* (**C**), *Tas2r135* (**D**), *Tas2r137* (**E**), and *Tas2r143* (**F**) was visualized by accumulation of black grains in radioactive in situ hybridization (ISH) experiments using anti-sense (as) riboprobes. ISH signals were absent from vallate papilla taste buds in mice lacking expression of *Pou2f3* (Pou2f3^–/–^) (**G–L**). Tuft cells in the mucosal epithelium of the gallbladder from *Pou2f3*^+*/*+^ mice were detected by presence of *Pou2f3* mRNA (**M**, arrows). No detectable expression was found for *Tas2r108* (**N**), *Tas2r126* (**O**), *Tas2r135* (**P**), *Tas2r137* (**Q**), and *Tas2r143* (**R**). Likewise, no ISH signals were present in tissue samples from the gallbladder taken from *Pou2f3*^*–/–*^ mice (**S–X**). Sections were counter-stained with methyl green. The luminal side (l) is always displayed up. e, epithelial layer; m, muscle layer. The scale bar in (**A**) equals 100 µm and applies to images A-K. The scale bar in (**M**) equals 50 µm and applies to images (**M–X**).

Next, we studied cellular localization of *Tas2r* mRNA expression by ISH. Guided by the results from the RT-PCR and the in-silico analysis of scRNA sequencing data, we generated gene-specific riboprobes for those *Tas2r* family members which had been consistently found by these techniques (*Tas2r108*, *Tas2r126*, *Tas2r135*, *Tas2r137*, *Tas2r143*). Initially, the sensitivities and specificities of all riboprobes were assessed in sections containing vallate papilla taste buds taken from *Pou2f3*^+*/*+^ and from *Pou2f3*^*–/–*^ mice that lack type II taste cells^38^. As expected, *Pou2f3* mRNA was detected by ISH in taste buds from *Pou2f3*^+*/*+^, but not from *Pou2f3*^*–/–*^ mice (Fig. 7). Likewise, transcripts for components of the canonical bitter taste transduction cascade^39^, namely Gnat3, Plcb2, and Trpm5 were present in vallate papilla from *Pou2f3*^+*/*+^, but not from *Pou2f3*^*–/–*^ mice (Sup. Fig. 7). To prove that taste buds were still present in *Pou2f3*^*–/–*^ mice, expression of the type II + III taste cell structural marker, *Vil1*, was examined. *Vil1* gene expression was found both in *Pou2f3*^+*/*+^^37^, and in *Pou2f3*^*–/–*^ taste buds (Sup. Fig. 7). Sense probes for all transcripts never resulted in specific staining in ISH experiments (Sup. Figs. 7 + 8). When testing riboprobes for the above mentioned five *Tas2r* genes in ISH experiments, we observed signals for all genes in taste buds from *Pou2f3*^+*/*+^ mice, but not in taste buds from *Pou2f3*^*–/–*^ mice (Fig. 7), indicating expression restricted to type II taste cells in the vallate papilla. Again, use of sense riboprobes did not result in specific staining (Sup. Fig. 8). Next, we assessed the expression patterns of *Pou2f3*, *Tas2r108*, *Tas2r126*, *Tas2r135*, *Tas2r137,* and *Tas2r143* in the mouse gallbladder by ISH. The gallbladder wall consists of a mucosa, a muscle layer, and a serosa^1^. Besides columnar gallbladder epithelial cells, the mucosal epithelial layer harbors tuft cells, which express and depend on *Pou2f3* (Fig. 7M, S). Probing for *Tas2r108* (Fig. 7N, T), *Tas2r126* (Fig. 7O, U), *Tas2r135* (Fig. 7P, V), *Tas2r137* (Fig. 7Q, W), and *Tas2r143* (Fig. 7R, X) did not result in detectable signals in any of the three layers, neither in *Pou2f3*^+*/*+^nor in *Pou2f3*^*–/–*^ mice.

**Figure 8:**
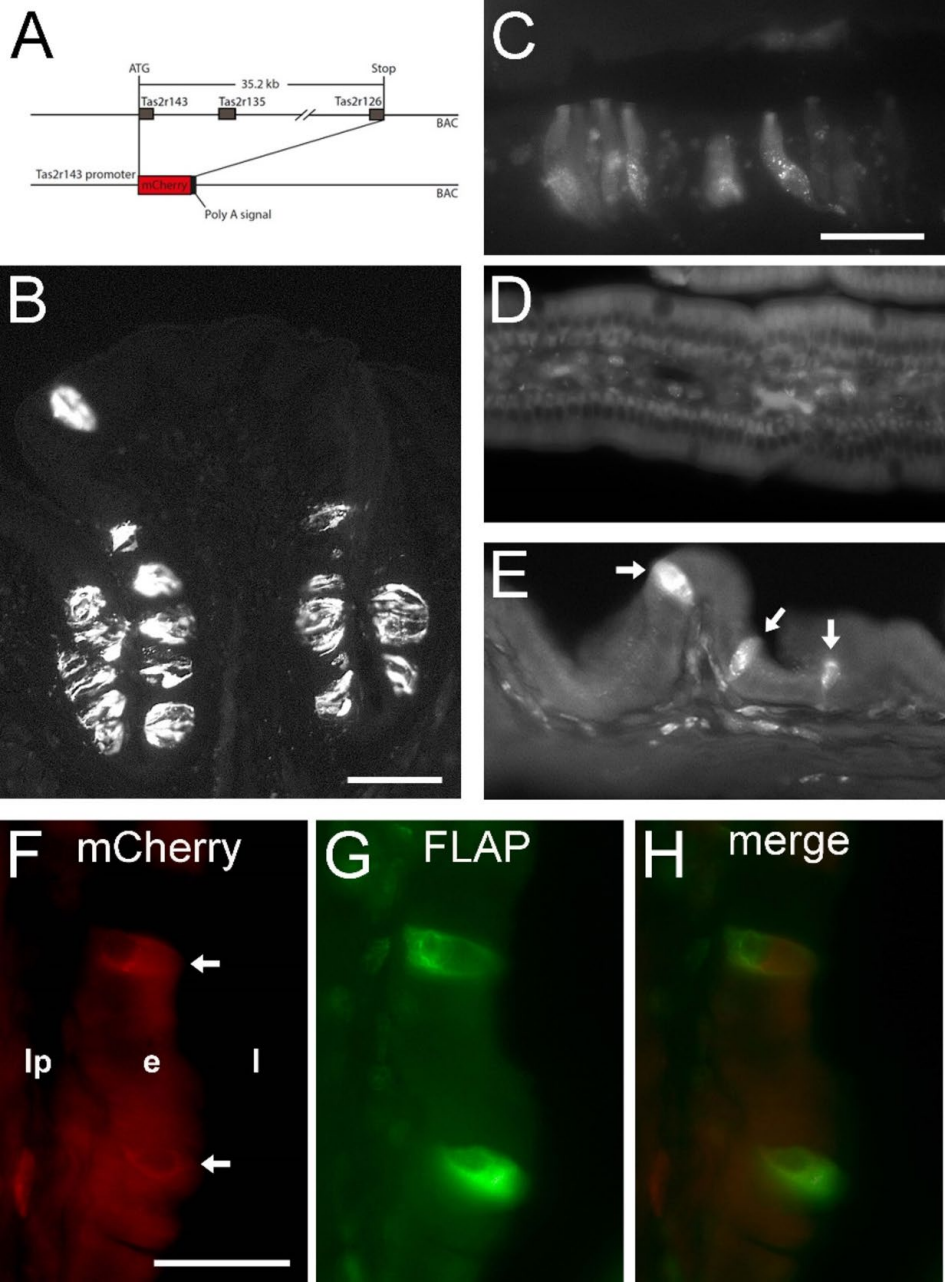
Expression pattern of a TAS2R143-mCherry reporter mouse line. (**A**) Scheme depicting the generation strategy of the TAS2R143-mCherry transgenic mouse line. Native mCherry fluorescence is detectable in sections from vallate papilla taste buds (**B**), in epithelial cells at the squamo-columnar junction in the stomach (**C**), and in solitary cells in the gallbladder epithelium (**E**), but is absent from the epithelium of the duodenum (**D**) (representative images from *n* = 4 mice). Note some unspecific labeling of cell nuclei in lamina propria. Immunofluorescence for mCherry [arrows in (**F**)] overlaps with FLAP signals (**G**) in the gallbladder (**F–H**). The scale bar in (**B**) equals 100 µm, the scale bar in (**C**) equals 50 µm and also accounts for (**D,E**). The scale bar in (**F**) equals 25 µm and also accounts for (**G,H**).

Finally, we took advantage of a newly generated reporter mouse line that expressed the fluorophore mCherry under the control of the *Tas2r143* promoter (TAS2R143-mCherry) (Fig. 8A). When analyzing tissue sections from this mouse line for mCherry fluorescence, we observed intense fluorescent labeling of vallate papilla taste buds (Fig. 8B), columnar cells at the squamo-columnar junction in the stomach (Fig. 8C), and single cells in the gallbladder mucosal epithelium (Fig. 8E), but not in the small intestine, i.e. duodenum (Fig. 8D). Double-fluorescence analysis for mCherry and 5-lipoxygenase-activating protein (FLAP), a marker for tuft cells, showed that expression from the TAS2R143-mCherry transgene was confined to tuft cells in gallbladder sections (Fig. 2F–H).

## Discussion

This study demonstrates profound relaxing effects of some prototypic bitter tastants on the mouse gallbladder, together with the expression of a select panel of *Tas2r* and of members of the canonical taste transduction signaling cascade in this organ, but does not support the concept of a causal link between them, i.e. bitter receptor-mediated relaxation.

In general, bitter receptor expression appeared to be widespread, although not ubiquitous among cell types in the murine gallbladder. A limited cell type specificity was noted for *Tas2r108* and *Tas2r137*, whose expression was largely reduced, albeit not fully abrogated, in *Pou2f3*^*–/–*^ mice lacking tuft cells. At the single cell level, neither in gallbladder tuft cells, nor in other cell types their expression was high enough to be detected by ISH, despite strong signals were obtained in taste buds, and only one positive cell showed up in the scRNA-seq data set. TAS2R108 and TAS2R137 can be activated by quinine and denatonium^7,9^, two of the bitter tastants used in this study, and we recently identified tuft cells as potential regulators of gallbladder tone^3^. Hence, activation of TAS2R108 and TAS2R137 on tuft cells appeared as one possibility how bitter tastants might exert effects on muscle tone. However, the relaxant response to quinine remained fully intact in *Pou2f3*^*–/–*^ mice, excluding a role of tuft cells and making an involvement of TAS2R108 and TAS2R137, in view of their minimal residual expression in these mice, unlikely.

The genes of the other three bitter receptors consistently detected by RT-PCR, *Tas2r126, Tas2r135* and *Tas2r143*, are grouped in a cluster on mouse chromosome 6, which may facilitate co-expression. Still, expression of only *Tas2r126* and not of the others was noted in the mouse stomach in a previous study utilizing another *Tas2r143*-reporter strain^40^. Expression of these three *Tas2r* was not reduced in gallbladders of *Pou2f3*^*–/–*^ mice, indicating that non-tuft cells contribute most to their transcript levels in the gallbladder. Accordingly, scRNA-seq data analysis revealed hits scattered across two (*Tas2r126*), six (*Tas2r135*) and seven (*Tas2r143*) different cell clusters. Again, only few cells per cluster were positive for all these receptors, and transcripts of these genes were undetectable at single cell level by ISH. Consistent with some *Tas2r143* hits in the main epithelial cell cluster, single epithelial cells were noted in the TAS2R143-mCherry reporter mouse line. These cells, however, were found FLAP-immunoreactive and thus tuft cells. Transcriptional activation of this gene cluster thus may be more complex or selective than previously suggested, mRNA stabilities may vary, or their expression level was underestimated in the scRNA data set due to inherent limitations. Nevertheless, the TAS2R143-mCherry reporter mouse line introduced in this study represents a new tool to study bitter taste-related tuft cell biology in mice.

Among this group of bitter receptors, the ligand profile of TAS2R143 is largely unknown, but TAS2R126 can be activated by quinine and TAS2R135 by denatonium^7^, making this cluster a candidate for causing the relaxant response observed in this study. Indeed, quinine relaxed the pre-contracted gallbladder, but genetic deletion of the entire gene cluster had no impact on this quinine-induced relaxation, similar to what has been reported for mouse bronchi^41^.

The possibility that low-level expression of other TAS2R, including those that were not analyzed by ISH, also linked to the canonical signaling pathway, may account for the relaxant responses led us to interrupt the canonical taste transduction cascade at the level of the critical ion channel TRPM5 (*Trpm5*^–/–^). Again, this left the responses unaffected, still leaving the possibility that TAS2R with low-level expression utilize intra-cellular signaling pathways that are different from the classical cascade. Along this line, Woo et al. recently demonstrated Tas2R-mediated signaling inducing relaxation of human airway smooth muscle through destabilizing filamentous actin and dephosphorylating cofilin^27^.

Tuft cells were not required, because the relaxing effect of quinine was found unchanged in gallbladders lacking tuft cells (*Pou2f3*^–/–^). This finding argued for direct actions of bitter tastants on gallbladder SMC, which was confirmed by recordings of [Ca^2+^]_i_ in isolated cells. Quinine and DXM preferentially, but not exclusively increased [Ca^2+^]_i_ in the gallbladder SMC population. Intriguingly, the maximum calcium response in presumed SMC was significantly higher in C57BL/6 mice, when compared to wildtype and knockout littermates of our TRPM5-deficient mouse line. Responses to CCK stimulus in SMC taken from *Trpm5*^*–/–*^ mice, on the other hand, were not different from *Trpm5*^+*/*+^ littermates, highlighting that the selection of appropriate controls is central to these experiments. Since TRPM5 is not expressed by gallbladder SMC, a decreased calcium response in *Trpm5*^*–/–*^ mice may be attributable to effects by the genetic background of this mouse line, but not by the knockout itself.

Similar paradoxical [Ca^2+^]_i_-increasing effects have been observed for airway smooth muscle cells in culture and localized compartmentalization was considered as the reason why this did not translate into contraction^12^. In a subsequent study, Zhang and colleagues proposed a model where global [Ca^2+^]_i_ increases occur in resting cells to an extent that is not sufficient to cause contraction, but may reverse the increase in [Ca^2+^]_i_ evoked by bronchoconstrictors, ultimately leading to bronchodilation^34^.

Since we did not observe *Tas2r* expression in SMC in ISH experiments, low-level expression of *Tas2r* may be sufficient, or, more likely, bitter tastants elicit their activity on SMC in a TAS2R-independent way. Collectively, the genetic models used in this study strongly argue against a role of those five TAS2R family members, for which we regularly found mRNA expression, in the relaxant responses to the bitter tasting compounds tested, pointing towards effects being mediated through other pathways than bitter receptors^3,21,22,32,42^. Also, alternative modes of action are known for all bitter compounds used in this study. For example, dextromethorphan is an agonist at sigma-1 receptors and an antagonist at NMDA receptors^43^. Quinine can cause activation of RalA, a RAS p21-related small G protein^44^ and denatonium has toxic effects on mitochondria^45^. Of course, formally it cannot be excluded that other TAS2R family members with low mRNA abundancy and long half-life at protein level may have escaped detection and drive these responses.

## Materials and methods

### Mouse strains and handling

C57BL/6JRj mice were purchased from Janvier Labs (stock number #5751862). *Chat*^*BAC*^-eGFP (#007902), and *Trpm5*^*–/–*^ mice (#005848) were obtained from The Jackson Laboratory (Bar Habor, ME, USA). *Pou2f3*^*–/–*^ mice^38^ were obtained from the Monell Chemical Senses Center (Philadelphia, PA, USA). *Tas2r143/135/126*^*–/–*^ mice^32^ were obtained from the Max Planck Institute for Heart and Lung Research (Department of Pharmacology, Bad Nauheim, Germany). Transgenic mice expressing the fluorophore mCherry under the control of the *Tas2r143/135/126* promoter (TAS2R143-mCherry) were generated using the BAC clone RP23-316O11 (CHORI, CA, USA) from mouse chromosome 6 containing the *Tas2r143, Tas2r135, and Tas2r126* genes. The genomic sequence between the start codon of *Tas2r143* and the stop codon of *Tas2r126* (35.2 kb) on the BAC was replaced by a cassette carrying the mCherry cDNA followed by a polyadenylation (pA) signal and an FRT-flanked ampicillin resistance cassette using Red/ET recombination kit (Gene Bridges, Heidelberg, Germany) (Fig. 2). Correct targeting was verified by restriction digests and DNA sequencing. After Flp-mediated excision of the ampicillin resistance cassette and linearization, the recombined BAC was injected into C57BL/6 oocytes. Transgenic offspring was genotyped for BAC insertion by genomic PCRs. One founder was used to generate the *Tas2r143* reporter line. Animals were kept on a C57BL/6 background. For genotyping by PCR, the following primers were used: forward: 5′-caggagtcattgaactgggag-3′; reverse: 5′-aggatgtcccaggcgaagg-3′; PCR products of 423 bp indicated the transgenic allele.

All transgenic and corresponding wildtype or heterozygous littermates were housed in groups of 2–6 individuals under specified pathogen-free conditions. They were kept on a 12 h light/12 h dark cycle and had access to food and water ad libitum. The genotypes of all mice were verified by PCR using genomic DNA obtained from ear biopsies, according to published protocols supplied by the vendor (The Jackson Laboratory) or donators (I. Matsumoto, Tokyo, Japan, for *Pou2f3*^*–/–*^ mice; S. Offermanns, Bad Nauheim, Germany, for *Tas2r143/135/126*^–/–^ and *Tas2r143*-mCherry mice). Animals were anesthetized by isoflurane (5%) (Abbott) or carbon dioxide and euthanized by transecting the inferior vena cava.

All animals and all experimental protocols were handled in accordance with the guidelines established by the European Community for the care and use of animals and were approved by the local committee at the Regierungspräsidium Giessen, Hesse, Germany. Breeding and use of samples of euthanized mice for further experiments were registered by local animal welfare officers, and the responsible authorities at the Regierungspräsidium Giessen and Darmstadt (Hesse), Germany (registration numbers Ex-15-2016, 571_M, 573_M, 624_M, 641_M, 692_M, 693_M, 793_M). All experiments and methods were in compliance with ARRIVE guidelines.

### RT-PCR

Whole gallbladder and a piece of tongue containing the vallate papilla were shock-frozen in RLT buffer plus (51304, QIAamp DNA Mini Kit, Qiagen, Hilden, Germany) and stored at -80^◦^C until use. Total RNA was isolated by using the RNeasy kit according to the manufacturer’s protocol (Qiagen). Genomic DNA contamination was eliminated using DNase-I (79254, Qiagen) and a reverse transcription was done for 50 min at 42^◦^C using quantiscript reverse transcriptase (205313, Qiagen), with random hexamer primer. The subsequent PCR was performed by adding 1 μl cDNA, 0.5 μl of each primer (20 pM; MWG Biotech, Ebersberg, Germany) (Supplemental Table 1), 2.5 μl 10× PCR buffer II (100 mM Tris–HCl, 500 mM KCl, pH 8.3), 2 μl MgCl_2_ (15 mM), 0.5 μl dNTP (10 mM each), 0.1 μl AmpliTaq Gold polymerase (5 U/μl; all reagents from Applied Biosystems, Darmstadt, Germany) and 17.9 μl H_2_O. The cycling conditions were 12 min at 95 °C, 40 cycles with 30 s at 95 °C, 30 s at 59 °C, 30 s at 72 °C, and a final extension at 72 °C for 7 min. Control reactions included the absence of DNA template and the absence of reverse transcriptase. The PCR products were separated by gel electrophoresis on a 2% TRIS–acetate-EDTA agarose gel with a 100 bp DNA ladder (15628050, Invitrogen, Karlsruhe, Germany) as length marker, and finally detected by UV illumination using a spectrophotometer (Ultrospec 2100 Pro, Biochrom, Cambridge, UK).

Real-time PCR (iCycler, Bio-Rad, Germany; iTaq Universal SYBR Green Supermix, Bio-Rad Laboratories Inc., CA, USA) was conducted on gallbladders and tongues from *Pou2f3*^+/+^ and *Pou2f3*^*−/−*^ mice to quantify *Trpm5, PLCβ2, Gnat3, Tas2r108, Tas2r126, Tas2r135 and Tas2r143* expression, taking *β2-microglobulin* (*B2m*) as housekeeping gene. PCR primers are specified in Supplemental Table 1. The PCR conditions included initial denaturation for 10 min at 95 °C followed by 45 cycles of 30 s at 95 °C, 30 s at 61 °C, and 30 s at 72 °C for *Trpm5*, and initial denaturation for 3 min at 95 °C followed by 45 cycles of 15 s at 95 °C, 15 s at 61 °C, and 15 s at 72 °C for the other targets. Relative expression was calculated as 2^−ΔCT^, with CT = cycle threshold and ΔCT = CT (target) − CT (*β2m*).

### ScRNA data set analysis

A mouse gallbladder gene expression dataset was downloaded from GEO datasets at NCBI (GSM5888949) and re-analyzed. The Seurat R package (version 2.3.4) was employed for analysis and re-clustering, utilizing principal component analysis for initial assessment. Subsequently, Uniform Manifold Approximation and Projection (UMAP) was applied for non-linear dimensional reduction, and cells were visualized in a two-dimensional UMAP plot. Cluster identification and annotation were based on typical marker genes. After the initial clustering, to explore the gene expression patterns within each identified cluster, violin plots were created by using the Seurat R package (version 2.3.4) and its built-in functions for plotting. The data, however, comes with inherent limitations. The dataset was generated using short reads and exhibited a relatively low sequencing depth. This aspect of the experimental design has the potential to lead to an underrepresentation of low-expressed genes, including those encoding G protein-coupled receptors (GPCRs). GPCRs, known for their involvement in various physiological processes, often demonstrate lower expression levels compared to other genes. To complement the experimental findings, an in-silico approach was employed. The in-silico method involved computational simulations, leveraging predictive algorithms to analyze the transcriptome dynamics of gallbladder cells. This computational perspective enhanced the comprehensive examination of the dataset. Despite the robustness of the in-silico method, it is essential to acknowledge that computational analyses also have inherent limitations, and results should be interpreted in the context of these considerations.

### In situ hybridization

For in situ hybridization (ISH), the vallate papilla was identified at the back of the tongue and removed with surrounding muscle tissue. The gallbladder was left attached to a piece of liver tissue during dissection to minimize the risk of damage. The tissue used for ISH was submerged in TissueTek OCT compound (Sakura Finetek Europe B.V, Alphen aan den Rijn, The Netherlands) and quickly frozen in isopentane (2-methylbutane, 78-78-4, Sigma-Aldrich, Steinheim, Germany) at −40 °C. After an initial storage at −70 °C, 16 µm thick tissue sections were cut at −15 °C using a cryostat (CM3050S, Leica Microsystems, Nussloch, Germany) and mounted on silanized (3-(Triethoxysilyl)-propylamine, 919-30-2, Merck Millipore, Darmstadt, Germany) glass slides. Tissue sections on glass slides were air-dried at room temperature for 30 min and subjected to the following procedure (all procedures at room temperature unless otherwise stated): 1 h fixation in freshly prepared 4% (w/v) paraformaldehyde (PFA) in phosphate-buffered saline (10 mM PBS, pH 7.5), three times 10 min each washing in PBS, permeabilization for 10 min in 0.4% (v/v) Triton X-100 (9002-93-1, Carl Roth) in PBS. After additional washes in PBS, the sections were acetylated for 10 min with triethanolamine/acetic anhydride (Sigma-Aldrich), washed again, dehydrated in 50% and 70% isopropanol, and finally air-dried. Complementary anti-sense (as) RNA probes for the detection of mouse transcripts in tissue sections were generated in the following way: first, gene-specific DNA fragments for *Gnat3* (GeneBank acc. no. NM_001081143.1, nucleotides (nt) 86–840), *Plcβ2* (NM_177568.2, nt 309–1192), *Pou2f3* (NM_011139.2, nt 110–921), *Trpm5* (NM_020277.2, nt 461–1292), *Villin* (*Vil)* (NM_009509.2, nt 1531–2318), *Tas2r108* (NM_020502.1, nt 44–848), *Tas2r126* (NM_207028, nt 68–879), *Tas2r135* (NM_199159.1, nt 147–946), *Tas2r137* (NM_001025149.1, nt 193–1000), and *Tas2r143* (NM_001025061.1, nt 80–795) were amplified by PCR (for primer and amplicon length details see Supplemental Table 1) using mouse C57BL/6J ileum cDNA as template, and subcloned into pGEM-T (pGEM-T Vector System, Promega, Mannheim, Germany). The identity of the cloned gene fragments was confirmed by double-stranded sequencing (Microsynth Seqlab GmbH, Göttingen, Germany). Antisense and sense riboprobes were generated by in vitro transcription using T7 RNA Polymerase (Sigma-Aldrich) and SP6 RNA polymerase (Roche Diagnostics, Mannheim, Germany), respectively, and radioactive (UTPαS [35S], NEG039C001MC, PerkinElmer, Waltham, USA)-labeled nucleotides. The ISH procedure was essentially performed as described previously^36^, with a few modifications. The tissue sections on microscopic slides were covered with 30–40 μl of hybridization solution, containing 50% formamide (75-12-7, VWR International S.A.S, Briare, France), 0.6 M NaCl (AppliChem, Darmstadt, Germany), 10 mM Tris/HCl (pH 7.4) (Carl Roth), 1 mM Na_2_EDTA (Carl Roth), 1× Denhardt’s solution (Sigma-Aldrich), 10% dextran sulfate (dextran sulfate sodium salt from *Leuconostoc* spp., Sigma-Aldrich), 100 mg/ml sonicated salmon sperm DNA (Agilent, Santa Clara, USA), 0.05% (w/v) *E. coli* MRE600 tRNA (Sigma-Aldrich), 20 mM dithiothreitol (3483-12-3, Roche), 50,000 d.p.m./µl S35-labeled riboprobe, and cover-slipped. Hybridization was carried out overnight at 60 °C in a humid chamber (Nunc Square BioAssay Dishes, Thermo Scientific). After hybridization, coverslips were removed in 2 × standard saline/sodium citrate (SSC) (20×: 3 M NaCl, AppliChem, 0.3 M Na_3_Citrate × 2 H_2_O, Carl Roth, 5 M HCl, Carl Roth) and the sections washed in the following order: 15 min in 2 × SSC, 15 min in 1 × SSC, 30 min at 37 °C in RNase solution [20 mg/ml RNase A (AppliChem) and 1 U/ml RNase T1 (Roche Diagnostics)], 30 min at RT in RNase-solution, 15 min in 1 × SSC, 15 min in 0.5 × SSC, 15 min in 0.2 × SSC, 60 min at 60 °C in 0.2 × SSC, 15 min in 0.2 × SSC, and finally 15 min in distilled water. For the visualization of radioactive hybridization signals, sections were exposed to Carestream BIOMAX MR autoradiography film (Sigma-Aldrich) for 2–3 days to estimate further exposure times, then coated under absence of light with nuclear emulsion (Ilford K5, Harman Tech., Mobberley, UK), exposed for 2 weeks at 4 °C in the dark and finally developed using Ilford Phenisol developer (Harman) and Ilford Hypam fixer (Harman). Sections were counterstained with methyl green (Sigma-Aldrich), and cover-slipped. Antisense and sense RNA probes were run in parallel in the same experiment to ensure equivalent conditions. In each experiment, vallate papillae containing taste buds were used as positive control.

### Immunohistochemistry

Immunohistochemistry (IHC) was performed according to two different protocols. On the one hand, freshly dissected gallbladders from a *Chat*^*BAC*^-GFP tuft cell-reporter mouse strain (see Sup. Fig. 6 upper row) were carefully opened with a surgical scissor, pinned on wax plates, and immersion fixed overnight in 4% PFA in 0.1 M phosphate buffer, washed, dehydrated and embedded in paraffin (Tissue-Tek Paraffin Wax, Sakura Finetek Europe B.V., Alphen aan den Rijn, The Netherlands). The specimens were cut at a thickness of 8 μm, mounted on silanized glass slides, and dried overnight at 37 °C. Sections were stored at room temperature until use. Before incubation with the primary antibodies, all sections were deparaffinized (xylene, 100%, 96%, 80%, 70% and 50% ethanol, respectively, each step for 5 min). Next, microwave oven heating for antigen retrieval was done for 10 min in PBS containing 10% citric acid, pH 6 (Merck, Darmstadt, Germany). The slides were incubated for 1 h in 10% normal horse serum, 0.5% Tween-20, 0.1% bovine serum albumin in PBS, pH 7.4, before applying the appropriate primary antibody combination overnight at room temperature. Primary antibodies included combination of rabbit anti-TPRM5 (1:200)^46,47^, and chicken anti-GFP (NB100-1614, 1:4000, Novus). After a washing step, the sections were incubated with secondary antibodies, Cy3-conjugated anti-rabbit IgG from donkey (1:2000, Merck Millipore) with FITC-conjugated anti-chicken IgY from Donkey (703-095-155, 1:2000, Dianova) for 1 h at room temperature. The slides were rinsed, postfixed for 10 min in buffered 4% PFA, rinsed again, and coverslipped with carbonate-buffered glycerol (pH 8.6). The sections were evaluated with an epifluorescence microscope (Axioplan Zeiss, Jena, Germany, Jena, Germany) using appropriate filter sets. The specificity of secondary reagents was validated by omission of primary antibodies.

Alternatively, whole gallbladder with a piece of surrounding liver tissue from C57BL/6J mouse strain was immersion-fixed overnight in Bouin Hollande fixative (see Sup. Fig. 6 lower row), then extensively washed in 70% isopropanol, dehydrated, and embedded in paraffin^48^. Paraffin blocks were cut with a microtome at 7 µm thickness, and mounted on silanized microscope glass slides. After deparaffinization and blocking procedures (see above), two primary antibodies from different donor species (rabbit anti-TRPM5, see above; and FITC-conjugated monoclonal mouse anti-α-smooth muscle actin (αSMA, 1:500, clone 1A4; Sigma-Aldrich, Taufkirchen, Germany, RRID: AB_476977) were co-applied in PBS/1% BSA and incubated over night at 16 °C, followed by 2 h at 37 °C. After extensive washing in distilled water followed by PBS, immunoreactions for TRPM5 were visualized by incubation for 2 h at room temperature with a secondary antibody labeled with Alexa Fluor 647 (chicken anti-rabbit-IgG, 1:200, Art.-No. A21443, Life Technologies). Immunofluorescence signals were documented in a surface scan using a BX50WI confocal laser scanning microscope (Olympus Optical, Hamburg, Germany) and Olympus Fluoview 2.1 software, and stored as false color images (8-bit tiff format) without modifications in brightness and contrast.

### Visualization of mCherry fluorescence

For the visualization of native mCherry fluorescence, selected tissues, i.e. gallbladder, tongue, stomach and small intestine were immersion fixed in 4% PFA for 4 h, washed several times in 0.01 M phosphate buffer, then cryo-protected by incubation overnight in the same buffer with 20% sucrose (Carl Roth), and frozen in TissueTek OCT compound by placing in isopentane cooled with liquid nitrogen. Sections (16 µm thick) were cut with a cryostat, mounted on glass slides, and cover-slipped using Fluoromount-G (Art.no. 15586276, Thermo Fisher Sci.). Native fluorescence was documented with a Zeiss Imager M2 microscope (Zeiss, Oberkochem, Germany), equipped with a Zeiss AxioCam HRc camera, and Zeiss ZEN 2011 software. To combine the visualization of native mCherry fluorescence with single immunofluorescence, a goat anti-5 lipoxygenase-activating protein (FLAP, NB300-891, 1:50; Novus Biol., Centennial, CO, USA) antiserum was applied in PBS/1% BSA and incubated overnight at 16 °C, followed by 2 h at 37 °C. After extensive washing in distilled water followed by PBS, immunoreactions were visualized by a two-step procedure using a species-specific biotinylated secondary antibody (703-065-155, 1:200, Jackson ImmunoResearch), applied for 45 min, followed by streptavidin conjugated with Alexa Fluor 488 (016-160-084, 1:200, Jackson ImmunoResearch) for 2 h.

### Organ bath force recording

Whole gallbladders were harvested and placed in minimum essential medium (MEM; Invitrogen Gibco, Oslo, Norway). They were mounted between two stainless steel clips in a computerized organ-bath chamber system (AD Instruments GmbH, Heidelberg, Germany). The chamber was filled with 37 °C warm MEM, which was supplemented with 1% penicillin/streptomycin (Sigma-Aldrich; P4333) and continuously aerated with a 95% O_2_/5% CO_2_ gas mixture. The temperature was held at 37 °C using a bath circulator (Thermo Fisher Scientific, Waltham, USA). The upper stainless clip was connected to an isometric force transducer (Power Lab 8.30; AD Instruments GmbH, Heidelberg, Germany) and converted signals were analyzed with LabChart 6 software (AD Instruments GmbH, Heidelberg, Germany). Changes in tension were recorded as force in gram (g). All gallbladders were first equilibrated against a passive load of 1 g and then adjusted at 0.5 g tension. All samples were equilibrated for 30 min until they reached a stable baseline tension. Pre-contraction was achieved by applying either 10 µM muscarine (2303-35-7, Sigma-Aldrich), or 0.1 µM cholecystokinin (CCK; fragment 26–33 amide, 25679-24-7, Sigma-Aldrich). For relaxation studies, dextromethorphan hydrobromide monohydrate (DXM) (6700-34-1), denatonium benzoate (3734-33-6), quinine hydrochloride, and noscapine hydrochloride (912-60-7) were obtained from Sigma-Aldrich. Stock solutions of DXM and quinine were dissolved in 100% ethanol, whereas all other drugs were dissolved in deionized H_2_O, and all drugs were further diluted in water or MEM to the desired experimental concentration immediately before use. Each type of experiment was performed on samples from at least three animals. The exact number of animals is indicated in the graphs. The delta force in percent with respect to the maximum force in response to either muscarine or CCK was calculated for each experiment.

### Cell separation and intracellular calcium recording

Gallbladder was dissected, cut into small pieces, and enzymatically digested at 37 °C for 30 min with dispase (2 mg/mL; Sigma-Aldrich) in HBSS (Invitrogen), and 5 min in trypsin/PBS (1:1, Invitrogen). After centrifugation (60×*g*, 5 min) and mechanical dissociation by pipetting, cells were resuspended in PBS. Isolated cells were plated on coverslips in MEM supplemented with 1% penicillin/streptomycin and 5% fetal bovine serum (26140079, Thermo Fisher) for 30–60 min at 37 °C. Fluo-4-AM (F14201, Thermo Fisher) was mixed with pluronic F-127 (P3000MP, Invitrogen, 0.005%) by sonication and diluted to a final concentration of 5 μmol/l in tyrode III solution (8 mM CaCl_2_, 130 mM NaCl, 5 mM KCl, 1 mM MgCl_2_, 10 mM 4-(2-hydroxyethyl)piperazine-1-ethanesulfonic acid, *N*-(2-hydroxyethyl) piperazine-*N*′-(2-ethanesulfonic acid) (HEPES), 10 mM glucose, 10 mM pyruvic acid, and 5 mM NaHCO_3_). Plated cells were loaded with the fluorescent calcium indicator in tyrode III solution for 10–30 min at 37 °C. Intracellular calcium concentration ([Ca^2+^]_i_) was analyzed with a confocal laser-scanning microscope (Zeiss LSM 710) equipped with an argon laser providing excitation at 488 nm and recording emission at > 514 nm during continuous superfusion (3 ml/min) with tyrode solution. SMC were identified by their response to 1 µM CCK, and cells with at least 10% increase in [Ca^2+^]_i_ evoked responses were considered as responder SMC. Fluorescence intensities at the start of the recording period were set arbitrarily at 100%.

### Statistical analysis

Data in the graphs depicting time courses or dose responses are presented as mean ± standard error of the mean (SEM) or medians ± interquartile ranges (IQR). Statistical analyses were performed using GraphPad Prism 7 software (La Jolla, CA, USA). An assessment of the normality of the data was done using Kolmogorov–Smirnov normality test and revealed significant deviation from a normal distribution. Therefore, nonparametric tests, i.e. Mann–Whitney U-test or Kruskall-Wallis test followed by Dunn’s multiple comparisons test, were applied. The difference in relative frequencies of [Ca^2+^]_i_ responses was evaluated using the Chi-square test. Values for EC_50_of agonist responses were determined using non-linear regression sigmoidal curve analysis according to the Hill equation. Differences were considered as statistically significant when *p* < 0.05.

### Supplementary Information

Supplementary Information.

## Data Availability

Data and materials availability: All data needed to evaluate the conclusions in the paper are present in the paper or the Supplementary Materials. A mouse gallbladder gene expression dataset was downloaded from GEO datasets at NCBI (GSM5888949) and re-analyzed.
