# Supplementary material for: Bitter tastants relax the mouse gallbladder smooth muscle independent of signaling through tuft cells and bitter taste receptors
Source: Sci Rep. 2024 Aug 8;14:18447. doi: 10.1038/s41598-024-69287-6 (PMC11310472; doi:10.1038/s41598-024-69287-6)
Supplement: Supplementary file 1 — Supplementary Information. [file 41598_2024_69287_MOESM1_ESM.docx]

##### Bitter tastants relax the mouse gallbladder smooth muscle independent of signaling through tuft cells and bitter taste receptors

Maryam Keshavarz ^1,2,3,*^, Anna-Lena Ruppert ^4^, Mirjam Meiners ^1,2^, Krupali Poharkar ^1,2^, Shuya Liu ^5^, Wafaa Mahmoud ^1,2,6^, Sarah Winterberg ^4^, Petra Hartmann ^1,2^, Petra Mermer ^1,2^, Alexander Perniss ^1,2,7^,Stefan Offermanns ^2,8^, Wolfgang Kummer ^1,2,*^, and Burkhard Schütz ^4,*^

Affiliations:

^1^ Institute for Anatomy and Cell Biology, German Center for Lung Research, Justus Liebig University, Giessen, Germany.

^2^ Excellence Cluster Cardio-Pulmonary Institute, Justus Liebig University, Giessen, Germany.

^3^ present address: Anatomy and Cell Biology, Institute of Theoretical Medicine, Faculty of Medicine, University of Augsburg; Augsburg, Germany.

^4^ Institute for Anatomy and Cell Biology, Philipps-University, Marburg, Germany.

^5^ III. Department of Medicine, University Medical Center Hamburg-Eppendorf, Hamburg, Germany.

^6^ present address: Department of Anatomy, Faculty of Medicine, Jordan University of Science & Technology; Irbid, Jordan.

^7^ present address: Division of Allergy and Clinical Immunology, Jeff and Penny Vinik Center for Allergic Disease Research, Brigham & Women’s Hospital and Department of Medicine, Harvard Medical School, Boston, MA, USA.

^8^ Department of Pharmacology, Max Planck Institute for Heart and Lung Research, Bad Nauheim, Germany.

* Corresponding authors. Email to: maryam.keshavarz@med.uni-augsburg.de; wolfgang.kummer@anatomie.med.uni-giessen.de; schuetzb@staff.uni-marburg.de

**Supplementary material**

**Sup. Table 1** Oligonucleotide primer pairs used in RT-PCR analysis and for the generation of *in situ* hybridization (ISH) probes

| **A. RT-PCR** |  |  |  |
| --- | --- | --- | --- |
| **Gene**  accession number | **forward primer** | **reverse primer** | **amplicon length** [bp] |
| *Trpm5*  NM_020277.2 | TGAGGAACGACCTTTGGCTA | ACACGGATCTTGGTGGATGT | 183 |
| *β2m*  NM_ 009451 | TGGTGCTTGTCTCACTGACC | CCGTTCTTCAGCATTTGGAT | 160 |
| *Tas2r102*  NM_199153.2 | GGAAGCTTGGTGTTCTTGCTTGG | AGATCAGCTCGGTCCACATTGC | 127 |
| *Tas2r103*  NM_053211.1 | ACCCCATTCGCTGTGTCTTT | CAGGAAGGTTACCACAGTTTGC | 150 |
| *Tas2r104*  NM_207011.1 | GAGCGTTTGGTTTAGCACCAG | TCACAGCTAGCGGAAAGGAA | 164 |
| *Tas2r106*  NM_207016.1 | AATGTTTCCTGGCAGGTTTACC | TTGAGGCATACAACTGCATCTT | 163 |
| *Tas2r107*  NM_199154.2 | TCCCTGCGGTCACTCAATCATC | CAGTGCCTTCAAAGAGGCTTGC | 70 |
| *Tas2r108*  NM_020502.1 | TGGATGCAAACAGTCTCTGG | GGTGAGGGCTGAAATCAGAA | 158 |
| *Tas2r109*  NM_207017.1 | GTCAAATTCAGGTGTTAGGAAGTCAC | CAATCACTGCTTGCAAGGCT | 183 |
| *Tas2r110*  NM_199155.2 | AGCCTTAGGTTGATAGGAGGAAA | GGATCGGCACCTCAGACAAT | 153 |
| *Tas2r113*  NM_207018.1 | GAAGAATATGCAGCACACCGC | CCCAGAGCCCAGACAAACAAA | 186 |
| *Tas2r114*  NM_207019.1 | ATCCACAGTTGCTGTCTCCTG | GCCATGAGATAAGCAAGCACC | 210 |
| *Tas2r115*  NM_207020.1 | ACGTTCCCTTCAGCAGATCA | ATGCTGAGGCTGGTAGCAAA | 194 |
| *Tas2r116*  NM_053212.1 | CTTTTGCTGTGTCACTGGTCA | TCTGATGTGGGCCTTAGTGCT | 119 |
| *Tas2r117*  NM_207021.1 | CTTTTCGTTGTATTTTGTGAGGTTGT | CTGTCTCAGCTTCATGTCTCCTACA | 90 |
| *Tas2r118*  NM_207022.1 | AAGTTGCACAACGGTTGCAGTG | TCTCCACCGGTGACAGTCTTTG | 68 |
| *Tas2r120*  NM_207023.1 | ATGGCAAAGGATGTCAAGATCAG | ATGACCTGCTGGGTAGAAGGA | 182 |
| *Tas2r121*  NM_207024.1 | CACTAAAGCCCATGTGAATGCC | AGACCCAGTCGTGTTTTATGTG | 120 |
| *Tas2r122*  NM_001039128.1 | TGTGGCAAGCTCCATTCTTGA | ACCTCCACAATGACACACCAG | 163 |
| *Tas2r123*  NM_207025.1 | ACGGGAATCTTAGGACATGGA | CTCCTAGGCAAATGTGGGCA | 179 |
| *Tas2r124*  NM_207026.1 | AGTCTCTGGCTTGCTACAGCTC | AGCTTCCCAGAAGCATGTGGAC | 127 |
| *Tas2r125*  NM_207027.1 | CTCCGAAGACACCACCACAG | CACCACAGCACCAACAGAGA | 237 |
| *Tas2r126*  NM_207028.1 | GCAGTGTGTGGGATTGGTCAAC | TCCCGGAGTACTCAACCAGATG | 62 |
| *Tas2r129*  NM_207029.1 | TTGCAGATGCCCACATCAGAGTC | TGGCACAGAGTAGGACATAGGTG | 60 |
| *Tas2r130*  NM_199156.1 | TCCTTCCTGGCCCTGTTTG | TGAATGGCTTGAAGGATAGATTAGAG | 132 |
| *Tas2r131*  NM_207030.1 | ATCAACATGGCTTGCCACCTG | AGCACACCTCTCAATCTCCACTTA | 105 |
| *Tas2r134*  NM_199158.1 | GCCTGGGAAGTGGTAACCTACA | GTGTTGCTTAGTATCAGAATGGTGGA | 63 |
| *Tas2r135*  NM_199159.1 | GCCCTTTCAGAACTTCGGGA | CCTGCCACATCCAGTACCTG | 160 |
| *Tas2r136*  NM_181276.1 | GGGCAACTTGACTGGGAAGA | ATGTGGGTTGAAGCACTGGG | 195 |
| *Tas2r137*  NM_001025385.1 | AGTGGTTGTTGGGATGCTGT | GAGCAGCAGAAGGTAGGCAA | 229 |
| *Tas2r138*  NM_001001451.1 | TGCTATTCAGCTCGCCTGCTTC | TGGCTTGGTAGTTGTGGCTCAG | 62 |
| *Tas2r139*  NM_181275.1 | ACACACCCTGAACATGAGAAACA | GGCCTGCATATGAGCCTCTATG | 70 |
| *Tas2r140*  NM_021562.1 | CATCTGAAGAACATGCAACACAATG | GCAGGGCCTTAATATGGGCT | 73 |
| *Tas2r143*  NM_001001452.1 | TTCCCAGGCTGCTGGTTGTATC | AGTTCCCGGTGGCTGAAATGAC | 69 |
| *Tas2r144*  NM_001001453.1 | TGGTTTGCTGCTTGGCTCAATG | TCAGAAGGAACAGAGGGTGAGC | 73 |
| *Gnat3*  NM_001081143 | TCATCCATAAGAATGGTTACAGC | CCCACAGTCGTTTAATGATTTC | 231 |
| *PLCβ2*  NM_177568 | CCCACAGTCGTTTAATGATTTC | GGGAAGTCCTCTGGGTTGAT | 101 |
| **B. ISH** |  |  |  |
| *Gnat3*  NM_001081143.1 | CTGCCCAGCCACTAACATC | TGCATTCTGTTCACCTCCTC | 756 |
| *Plcβ2*  NM_177568.2 | CCGAGATACTCGCTTTGGGA | TAACTCTACACACCGGCAGC | 883 |
| *Pou2f3*  NM_011139.2 | ACTTTCGGTCAAGTGGAGCC | CTGCTCCGCAATCATGGAGA | 812 |
| *Villin-1* NM_009509.2 | GTCTATCTTCAAGGGCCGCA | CGTCCACTTTCGGGCTCATA | 788 |
| *Trpm5* NM_020277.2 | TGGCTAGCACATCCACCAAG | AAGAGGCGGACAAAGTCAGG | 832 |
| *Tas2r108* NM_020502.1 | TCGGCACCAAACGAGGAAAG | ATGGCCTGGGCTCTCAGATT | 805 |
| *Tas2r126* NM_207028 | CCAACGGCTTCATTGTGCTG | GCCGCGGAACCTGAGATTAT | 812 |
| *Tas2r135* NM_199159.1 | AGCACTGGGCATGAAATGGT | TCACCACTCTCAAAACAGGGT | 800 |
| *Tas2r137* NM_001025149.1 | ATTACCAGCTTGGCCCTCTTC | ATGAAGCAGAGGGTCCCTTAGA | 808 |
| *Tas2r143* NM_001025061.1 | TGATGATTGTGCTGGGCAGA | AGCCTCTCTGGCCCAATAGT | 716 |

**Sup. Table 2** Results of RT-PCR analysis of *Tas2r* expression in mouse tongue and gallbladder

| **Gene** | **To** | **Gb** | **Gene** | **To** | **Gb** |
| --- | --- | --- | --- | --- | --- |
| *Tas2r102* | 3/5 | 1/5 | *Tas2r123* | 2/3 | 1/5 |
| *Tas2r103* | 2/3 | 0/4 | *Tas2r124* | 2/3 | 1/4 |
| *Tas2r104* | 3/4 | 0/5 | *Tas2r125* | 1/3 | 1/5 |
| *Tas2r105* | 3/4 | 0/5 | ***Tas2r126*** | **5/5** | **6/7** |
| *Tas2r106* | 3/4 | 1/4 | *Tas2r129* | 2/3 | 1/3 |
| *Tas2r107* | 2/4 | 0/3 | *Tas2r130* | 1/3 | 0/3 |
| ***Tas2r108*** | **6/6** | **8/9** | *Tas2r131* | 2/5 | 1/7 |
| *Tas2r109* | 1/3 | 1/4 | *Tas2r134* | 2/3 | 1/4 |
| *Tas2r110* | 4/6 | 0/6 | ***Tas2r135*** | **4/4** | **7/8** |
| *Tas2r113* | 3/4 | 1/6 | *Tas2r136* | 2/3 | 0/4 |
| *Tas2r114* | 2/4 | 0/4 | ***Tas2r137*** | **2/3** | **7/7** |
| *Tas2r115* | 3/5 | 2/6 | *Tas2r138* | 2/6 | 3/6 |
| *Tas2r116* | 1/3 | 1/4 | *Tas2r139* | 1/3 | 1/4 |
| *Tas2r117* | 2/3 | 0/4 | *Tas2r140* | 1/3 | 1/4 |
| *Tas2r118* | 2/3 | 0/3 | ***Tas2r143*** | **3/4** | **5/6** |
| *Tas2r119* | 1/4 | 0/5 | *Tas2r144* | 2/3 | 0/5 |
| *Tas2r120* | 4/6 | 2/6 | ***Trpm5*** | **8/9** | **10/11** |
| *Tas2r121* | 1/3 | 0/3 | ***B2m*** | **8/8** | **8/8** |
| *Tas2r122* | 1/5 | 1/6 |  |  |  |

Values for tongue (To) and gallbladder (Gb) represent number of PCR-positive samples per total number of samples tested. *Tas2r* gene family members showing consistent expression in Gb (at least *n-1* positive samples per *n* tested samples) are highlighted. Expression of Trpm5 and β2MG served as positive control.


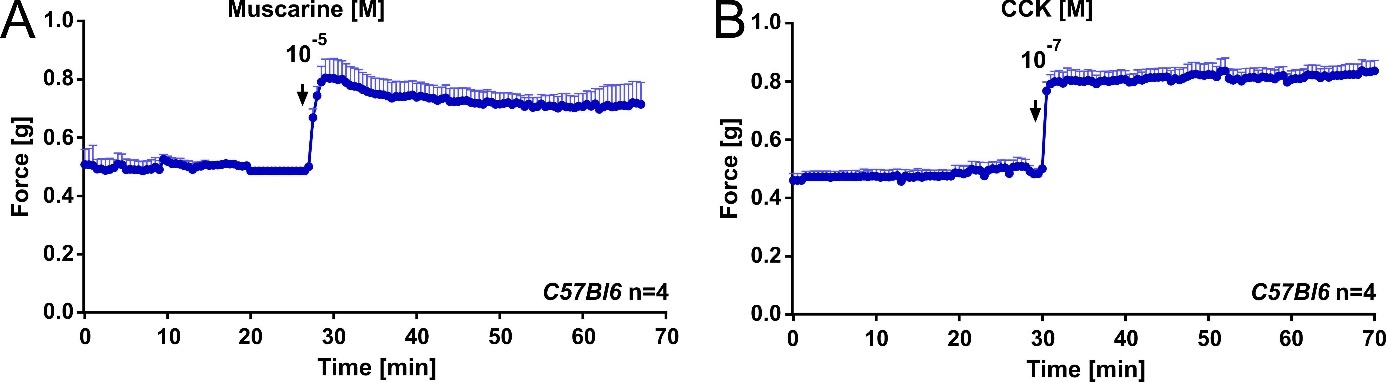


**Sup. Fig. 1** Muscarine and cholecystokinin (CCK) contract mouse gallbladder smooth muscle. Force recordings in organ bath experiments are displayed as mean ± S.E.M. (*n* = number of mice). S.E.M. is not displayed in the figure when the value is smaller than the symbol size. Note rapid and equal increase in force after stimulation with 10 µM muscarine (**A**), or 0.1 µM CCK (**B**). Also note persistence in contraction for additional 40 minutes after stimulation

**
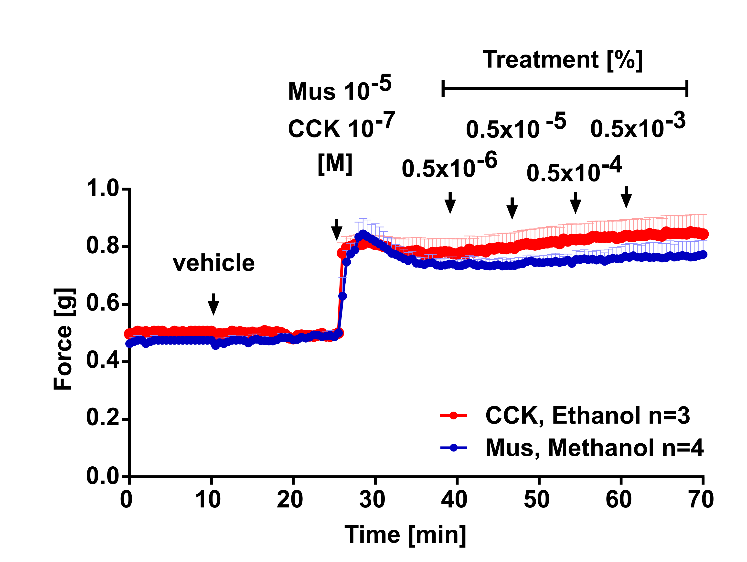
**

**Sup. Fig. 2** Vehicle controls for **TAS2R agonists have no effect on precontracted gallbladder.** Force recordings in organ bath experiments are displayed as mean ± S.E.M. (*n* = number of mice). S.E.M. is not displayed in the figure when the value is smaller than the symbol size. Control curves correspond to experiments in which only vehicle (ethanol or methanol) was added to the organ bath

**
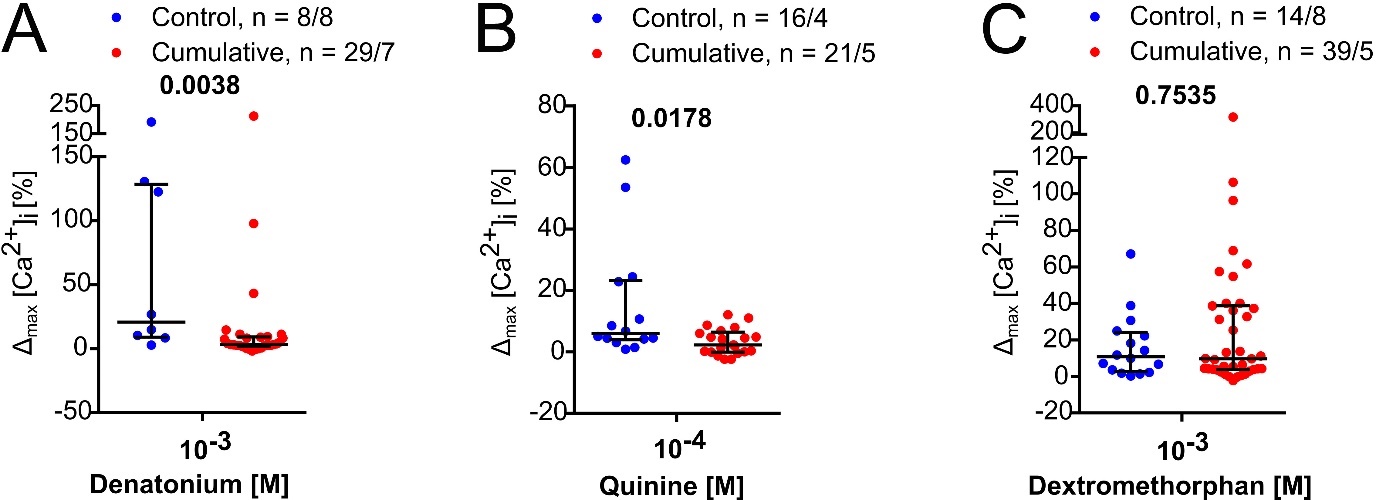
**

**Sup. Fig. 3** Calcium responses of gallbladder smooth muscle cells (CCK^+^) to denatonium and quinine desensitize. Maximum increase in [Ca^2+^]_i_ in CCK^+^ cells to the highest doses of (**A**) denatonium, (**B**) quinine, and (**C**) dextromethorphan used in this study given either directly after the initial CCK test stimulus (control) or at the end of the cumulative dose-response curves (cumulative) depicted in Fig. 5D-F. Cellular responses to denatonium and quinine were significantly lower at the end of the dose-response curves. Mann-Whitney-U-test, *p*-values are indicated; n refers to number of cells/number of gallbladders from which they were taken


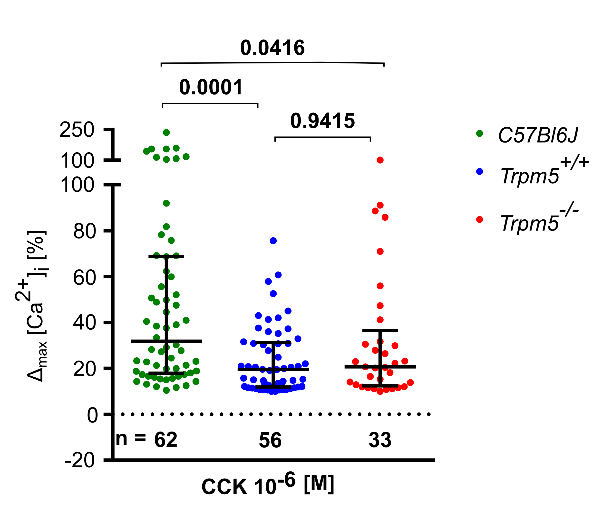


**Sup. Fig. 4** CCK^+^ cells from *Trpm5* wildtype and knockout mice are less responsive than cells from C57Bl6J mice. Maximum increase in [Ca^2+^]_i_ in CCK^+^ cells from C57Bl6J mice, *Trpm5* wildtype (*Trpm5^+/+^*) and gene-deficient (*Trpm5^-/-^*) mice. Reactions of *Trpm5^+/+^* and *Trpm5^-/-^* cells are not different from each other, but both significantly lower than reaction of C57Bl6J cells. Mann-Whitney-U-test, *p*-values are indicated; n refers to number of cells analyzed


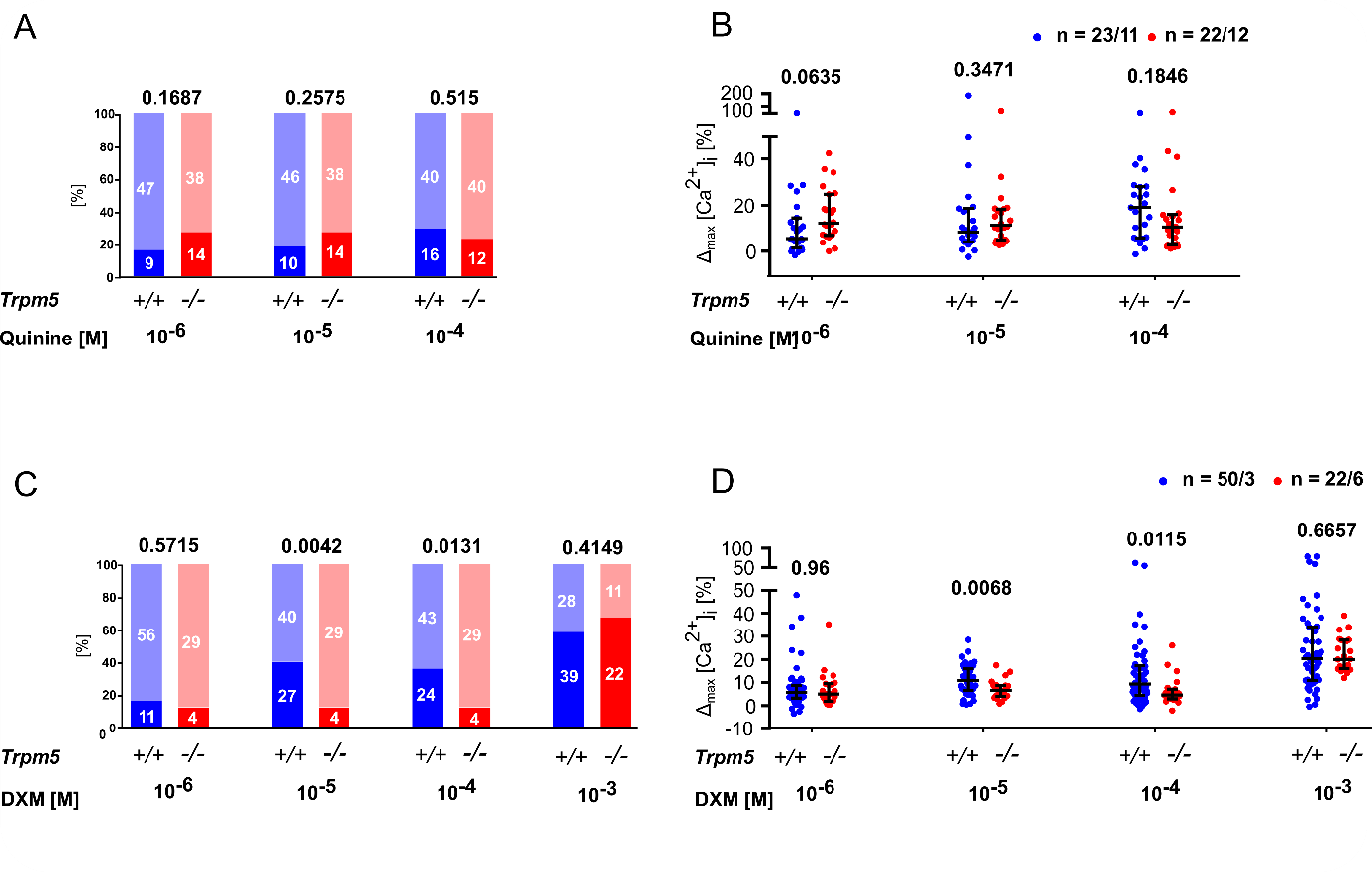


**Sup. Fig. 5** TRPM5 is not involved in [Ca^2+^]_i_ responses of isolated gallbladder CCK^-^-cells to quinine and dextromethorphan. Confocal laser scanning recordings of fluorescence intensity of the calcium indicator Fluo-4 in CCK^-^ cells. (**A, C**) Relative frequencies of responders (>10 % increase in [Ca^2+^]_i_; dark column) and non-responders (light column) to increasing concentrations of (A) quinine and (C) dextromethorphan (DXM) in cells from *Trpm5* wildtype (*Trpm5^+/+^*) and gene-deficient (*Trpm5^-/-^*) mice. Chi^2^-test shows no significant difference between genotypes, *p*-values indicated; absolute numbers of cells indicated in the columns. (**B, D**) Maximum increase in [Ca^2+^]_i_ in responders (cells that exhibited >10 % increase in [Ca^2+^]_i_ to at least one concentration of the respective bitter tastant) to (B) quinine and (D) dextromethorphan (DXM); extent of reaction is significantly different between genotypes (*Trpm5^+/+^* versus *Trpm5**^-/-^*) at DXM concentrations 10^-5^ and 10^-4^ M; Mann-Whitney-U-test, *p*-values are indicated; n refers to number of cells/number of gallbladders from which they were taken

**6**Immunohistochemical analysis of TRPM5-localization in the mouse gallbladder. In double-labeling immunofluorescence analysis, TRPM5 and ChAT (visualized by anti-GFP antibody) fully co-localize in solitary epithelial cells (upper row, marked by arrows) in a *Chat^BAC^*-GFP tuft cell-reporter mouse strain. Immunoreactivity for TRPM5 (lower row, marked by arrows) did not co-localize with immunoreactivity against alpha smooth muscle actin (αSMA) (C57BL/6J mouse strain). The bar in a equals 20 µm

**
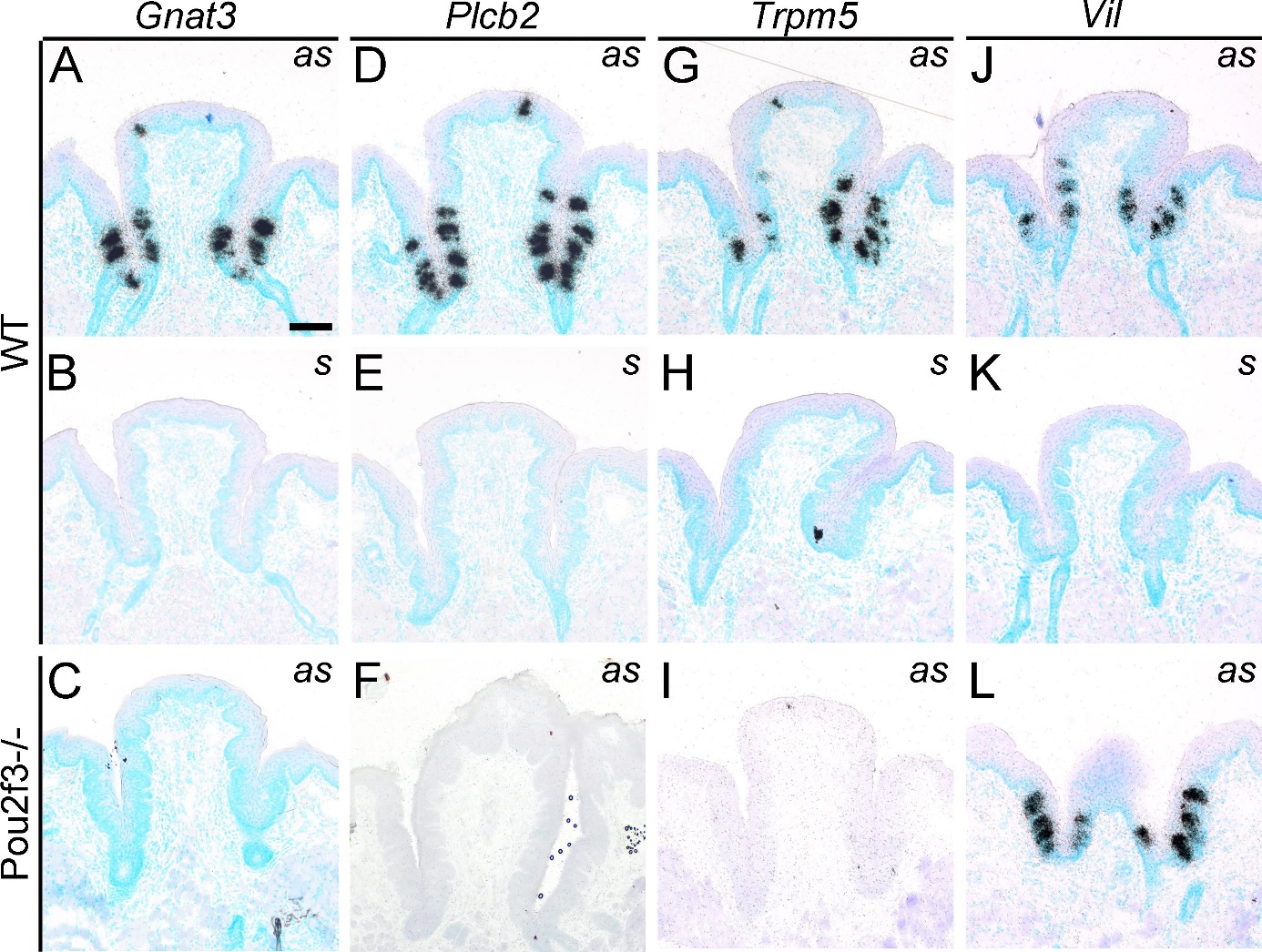
**

**Sup. Fig. 7** Gene expression patterns of tuft cell marker in taste buds from *Pou2f3^+/+^* and from *Pou2f3^-/-^* mice. Radioactive ISH with antisense (as) riboprobes revealed expression of members of the canonical taste transduction cascade, namely *Gnat3* (**A**), *Plcb2* (**D**), and *Trpm5* (**G**) in vallate papilla taste buds from *Pou2f3^+/+^*, but not *Pou2f3*^-/-^ mice (**C**, **F**, **I**), indicating expression in type II taste cells. *Vil* expression, on the other hand, was detected in both genotypes (**J**, **L**), indicating expression also in type III taste cells. Sense riboprobes did not result in specific labeling (**B**, **E**, **H**, **K**). The scale bar in a equals 100 µm and applies to all images


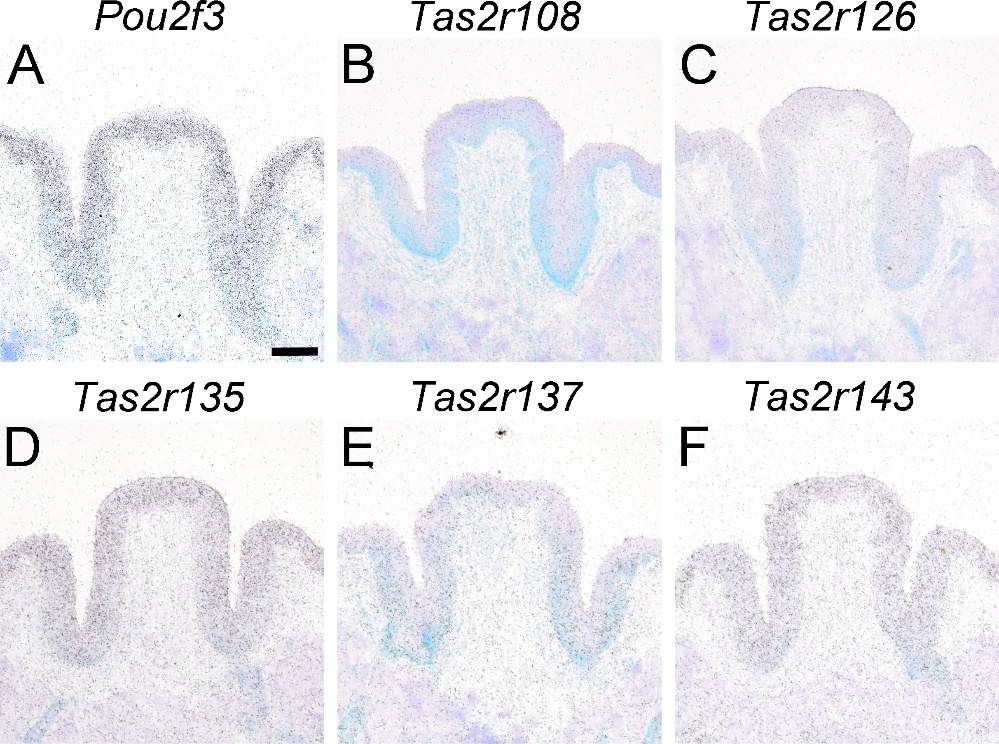


**Sup. Fig. 8** Sense riboprobe controls in ISH experiments. Depicted are tissue sections containing the vallate papilla, hybridized with sense riboprobes for *Pou2f3* (**A**), *Tas2r108* (**B**), *Tas2r126* (**C**), *Tas2r135* (**D**), *Tas2r137* (**E**), and *Tas2r143* (**F**). Note weak (B, C), moderate (E, F), and strong (A, D), however unspecific labeling, preferentially of the epidermis, in comparison to antisense riboprobes shown in Fig. 1. The scale bar in a equals 100µm and accounts for all images
